# Oral Pathogens’ Substantial Burden on Cancer, Cardiovascular Diseases, Alzheimer’s, Diabetes, and Other Systemic Diseases: A Public Health Crisis—A Comprehensive Review

**DOI:** 10.3390/pathogens13121084

**Published:** 2024-12-09

**Authors:** Peter E. Murray, Jonathan A. Coffman, Franklin Garcia-Godoy

**Affiliations:** 1 Independent Researcher, Fort Lauderdale, FL 33328, USA; p_e_murray@outlook.com; 2 College of Pharmacy, American University of Health Sciences, Signal Hill, CA 90755, USA; 3 College of Dentistry, University of Tennessee Health Science Center, Memphis, TN 38163, USA; godoy@uthsc.edu

**Keywords:** microorganisms, dental, antibiotics, teeth, caries, periodontitis, *P. gingivalis*, cardiovascular, antibiotic resistance

## Abstract

This review synthesizes the findings from 252 studies to explore the relationship between the oral pathogens associated with periodontitis, dental caries, and systemic diseases. Individuals with oral diseases, such as periodontitis, are between 1.7 and 7.5 times (average 3.3 times) more likely to develop systemic diseases or suffer adverse pregnancy outcomes, underscoring the critical connection between dental and overall health. Oral conditions such as periodontitis and dental caries represent a significant health burden, affecting 26–47% of Americans. The most important oral pathogens, ranked by publication frequency, include the herpes virus, *C. albicans*, *S. mutans*, *P. gingivalis, F. nucleatum, A. actinomycetemcomitans*, *P. intermedia*, *T. denticola*, and *T. forsythia*. The systemic diseases and disorders linked to oral infections, ranked similarly, include cancer, respiratory, liver, bowel, fever, kidney, complications in pregnancy, cardiovascular bacteremia, diabetes, arthritis, autoimmune, bladder, dementia, lupus, and Alzheimer’s diseases. Evidence supports the efficacy of dental and periodontal treatments in eliminating oral infections and reducing the severity of systemic diseases. The substantial burden that oral pathogens have on cancer, cardiovascular diseases, Alzheimer’s, diabetes, and other systemic diseases poses a significant public health crisis.

## 1. Introduction

The human body is home to a vast and diverse community of microorganisms, collectively referred to as the microbiome. Beginning with the microbes encountered in the fetal environment, these organisms significantly influence the development of human tissues and organs from early childhood onwards, playing a crucial role in shaping and regulating the immune system [1]. In children, the oral microbiome is primarily established through the vertical transmission of oral microbes from maternal saliva [2], although limited colonization from other caregivers can occur. The maternal microbiome is essential for “training” the immune system early in life by introducing diverse microbial signals [3]. In adulthood, microbes can either burden the human host with systemic diseases [4] or provide protection against infections, contributing to overall health [5]. There is an ongoing debate regarding whether pathogens initially colonize the mouth before migrating into systemic tissues, or if they first infect systemic sites and subsequently reach the mouth, or more likely, infect their host through multiple routes [6]. The acquisition and establishment of a healthy microbiota are crucial for a symbiotic host–microbiota relationship. The early exposure to specific microbes plays a pivotal role in developing a robust immune system that may have lasting effects on overall health [6].

**Figure 1:**
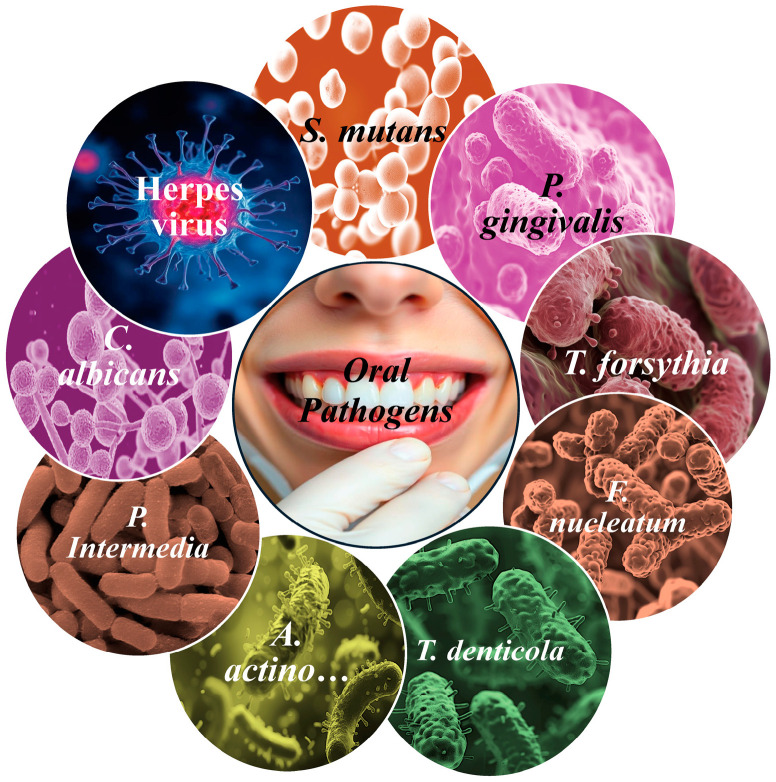
Oral pathogen morphology. The oral pathogens come in a variety of sizes and shapes, each adapted to their environment and function; most are anaerobic, non-motile, and are transmitted through saliva. Herpes viruses do not have a shape in the traditional sense since they are much smaller than bacteria or fungi and have a protein coat (capsid) surrounding their genetic material. *C. albicans* is a yeast with oval-shaped fungi that bud to reproduce. *S. mutans* is a coccus-shaped bacterium, because it has a round shape, and it typically forms pairs and chains of cocci. *P. gingivalis, F. nucleatum, P. intermedia*, and *A. actinomycetemcomitans* are bacillus (rod-shaped) bacteria. *T. forsythia* and *T. denticola* are spirochete (corkscrew-like)-shaped bacteria.

The human oral cavity hosts a complex microbiome of over 770 microbial species, although only a subset are pathogenic [7], including bacteria [8], fungi [9], and viruses [10] that can cause disease. The composition of the microbial communities varies among individuals and is influenced by factors such as diet, hygiene, genetics, and environmental exposure [11]. Unfavorable influences, including smoking, antibiotic use, and infections, can significantly alter the composition and structure of the oral microbiota, leading to oral and systemic diseases [12]. When oral microbiota dysbiosis occurs, certain commensal microorganisms in the mouth can become pathogenic, increasing in virulence and evading the immune defenses [13]. Beyond the oral cavity, humans host a diverse microbiota throughout the body, with the average adult microbiome weighing between 2 and 6 pounds and occupying approximately 3 pints in volume [14]. Most of the microbiota resides in the large intestine, which provides an expansive volume density for microbial colonization [14]. Oral pathogens come in a variety of sizes and shapes, each adapted to their environment and function, as shown in Figure 1.

Bacteria have existed on the Earth for at least 3.5 billion years, fungi for approximately 1 billion years, and viruses are estimated to have emerged around 450 million years ago. Fossil evidence suggests that these microorganisms were among the earliest life forms, appearing not long after the Earth stabilized enough to support life—billions of years before the emergence of humans. Their ancient origins highlight their fundamental roles in shaping the evolution of life on our planet [1,5].

**Figure 2:**
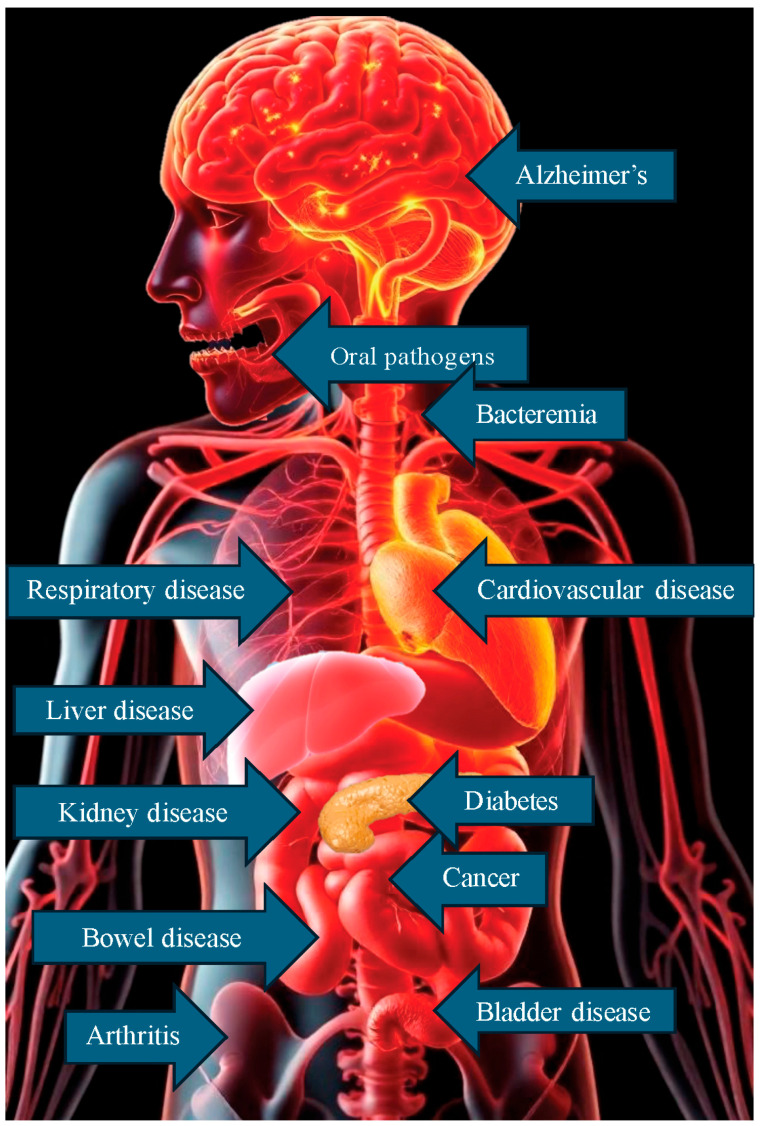
Diseases linked to oral pathogens. The oral pathogens can infect and damage multiple organs or selectively target specific ones, as organs offer an ideal environment with suitable levels of oxygen, carbon dioxide, nutrients, and protection from inflammatory responses and elimination processes.

The bacterial cell count in the human microbiome surpasses that of human cells [5], comprising nearly ten thousand distinct species [15]. Genetically, humans are much less diverse than their microbiota: while humans possess approximately twenty thousand genes, the collective human microbiome contributes around two million bacterial genes [16]. Oral pathogens can spread through the bloodstream, releasing pro-inflammatory cytokines and bacterial toxins like lipopolysaccharides (LPSs) that drive systemic inflammation [17]. This inflammatory response can contribute to chronic disease development and exacerbate the pre-existing health conditions in multiple organs and tissues throughout the human body [16], as shown in Figure 2.

## 2. Oral Pathogens

Oral pathogens are responsible for common diseases of the mouth including periodontal (gum) disease and dental caries (tooth decay) [18]. Common oral pathogens such as *P. gingivalis*, *A. actinomycete-mcomitans*, *T. forsythia*, and *T. denticola* have been identified as significant contributors to periodontal diseases [19]. Dental caries is caused by plaques, which are a community of microorganisms in a biofilm form [18]. 

**Figure 3:**
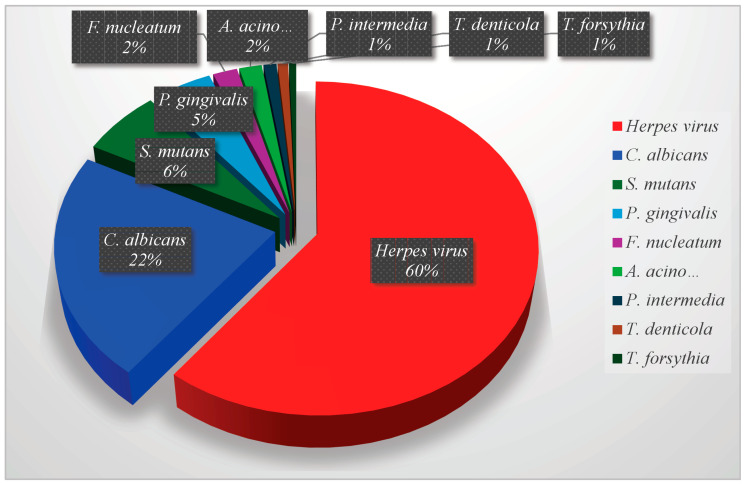
Pie chart of the important oral pathogens. The pie chart shows the most important oral pathogens, as proportion of total publications available by bacteria (oral pathogens) from a PubMed search conducted on 14 November 2024, with no time limit.

Genetic and peripheral factors lead to variations in the oral microbiome [20]. Homeostasis in the oral microbiome is preserved through commensalism and coexistence between microorganisms and the host [21]. However, cariogenic microorganisms cause dental caries, and other disorders and diseases, when the relationship with microorganisms becomes parasitic [22]. The oral microbiome of each person is quite distinct: while there are common species found in most individuals’ mouths; the specific composition of the oral microbiome can vary significantly from person to person [23]. Factors such as diet, oral hygiene practices, genetics, lifestyle choices (like smoking), age, health status, and even geographic location can all influence the unique microbial community in an individual’s mouth [24]. The individuality makes the oral microbiome as distinct as fingerprints, contributing to variations in oral health and susceptibility to diseases like caries, periodontitis, and other systemic conditions linked to oral health [25]. As a result, disease prevention strategies cannot be universally applied to all individuals. The risk of developing diseases is influenced by factors such as the composition of the oral microbiome, the presence and virulence of pathogens, and the immune system, which is in turn shaped by environmental and genetic factors [12]. The most significant oral pathogens [12] according to the proportions of publications in PubMed (Supporting Information) are the herpes virus (60%), *C. albicans* (22%), *S. mutans* (6%) *P. gingivalis* (5%), *F. nucleatum* (2%), *A. actinomycetemcomitans* (22%), *P. intermedia* (1%), *T. denticola* (1%), and *T. forsythia* (1%), as shown in Figure 3.

## 3. Herpes Viruses

Herpes Simplex Virus-1 (HSV-1), commonly known as oral herpes, is highly prevalent, with approximately 67% of the global population under the age of 50 carrying the virus [26]. Most infections are transmitted through oral-to-oral contact, particularly during childhood, often via kissing or sharing objects like utensils or towels [26]. Herpesviruses are a family of large, double-stranded DNA viruses that infect human cells and cause a wide range of diseases, from cold sores to severe systemic infections [27], Alzheimer’s disease [28], and multiple sclerosis [29]. Herpesvirus infections persist for the lifetime of the individual and may be associated with significant morbidity when they reactivate or are associated with malignancies [30]. In humans, the herpes family includes eight distinct viruses: Herpes Simplex Virus Type 1 (HSV-1) is often associated with oral herpes, causing cold sores around the mouth, although it can also cause genital infections [27]. Herpes Simplex Virus Type 2 (HSV-2) is primarily associated with genital herpes but can also infect the oral region. Varicella-Zoster Virus (VZV) causes chickenpox as a primary infection and can reactivate later in life to cause shingles [27]. Epstein–Barr Virus (EBV) is known for causing infectious mononucleosis (or “mono”) and is associated with some cancers, including Burkitt’s lymphoma and nasopharyngeal carcinoma [27]. Cytomegalovirus (CMV) typically causes mild symptoms in healthy individuals but can be serious in immunocompromised people and newborns [27]. Human Herpesvirus 6 (HHV-6) often causes roseola in infants, which is characterized by a high fever followed by a rash [27]; Human Herpesvirus 7 (HHV-7) is similar to HHV-6, often infecting children and causing mild illness. Kaposi’s Sarcoma-Associated Herpesvirus (KSHV or HHV-8) is associated with Kaposi’s sarcoma, a type of cancer that often affects immunocompromised individuals, such as those with AIDS [27].

Herpes viruses are among the largest and most intricate viruses known, having evolved over the past 400 million years [31]. Herpes viruses represent a highly successful evolutionary adaptation but constitute only a small fraction of all the possible herpes virus forms. Since they co-evolve with their hosts, the extinction of any host species also means the loss of its unique herpes viruses. Consequently, modern herpes viruses represent only a small remnant of all those that have existed throughout evolutionary history [31]. Due to the incurable nature of herpes viruses, research has primarily focused on developing vaccines that train the human immune system to recognize and respond to viral antigens. The objective is to enable the immune system to detect and neutralize the virus before it can infect host cells and cause an active infection. While vaccinations remain a subject of controversy due to the potential side effects, they are a critical tool in saving lives, protecting public health, and preventing herpesvirus infections, as well as reducing the risk of the associated preventable cancers [30].

## 4. *C. albicans*

Candidiasis is an opportunistic infection caused by *Candida*, a type of yeast commonly found in the oral cavity as part of the normal microbiota in 30–70% of people [32,33]. In healthy individuals, *Candida* typically remains harmless; however, in up to 90% of immunocompromised individuals—such as those with HIV/AIDS, diabetes, cancer patients undergoing chemotherapy, or those on immunosuppressive medications—*Candida* can proliferate excessively and cause oral thrush (oral candidiasis). Oral thrush is marked by white patches on the tongue, inner cheeks, gums, and the roof of the mouth, and can cause symptoms like pain, difficulty swallowing, and an unpleasant taste [34]. 

The human mouth hosts approximately 85 fungal species, with *Candida* as the most prevalent [35]. Fungi make up an estimated less than 0.1% of the total microbial population in the oral cavity. Current treatment options rely on antifungal drugs such as azoles, polyenes, and echinocandins but are delimited due to the emergence of drug-resistant strains and the associated adverse effects [36]. *C. albicans* is recognized for its role in polymicrobial infections, especially through its interactions with the other pathogenic bacterial species. Recent studies have highlighted how the polymicrobial biofilms enhance virulence by promoting mechanisms such as adhesion, invasion, quorum sensing, and the development of antimicrobial resistance [37]. Most fungal infections are superficial, commonly affecting the hair, skin, and nails. However, *Candida* species, particularly when originating from the oral cavity, can enter the bloodstream and spread throughout the body, infecting distant organs and tissues. The spread can lead to severe, potentially fatal systemic infections, especially in immunosuppressed individuals, if the *Candida* has developed a resistance to antifungal drugs [38].

## 5. *S. mutans*

*S. mutans* is often considered one of the most potent bacterial pathogens in the oral cavity, primarily due to its role in the development of dental caries (cavities) [39]. *S. mutans* is highly efficient at metabolizing sugars into acid, which demineralizes tooth enamel and contributes to tooth decay [40]. It is also capable of forming biofilms (plaque) on the teeth, which enhances its ability to colonize and persist in the mouth [41]. The bacterium’s ability to thrive in acidic environments and its production of extracellular polysaccharides helps it to adhere to tooth surfaces, making it a key player in dental caries [42,43].

Emerging research suggests that *S. mutans* is associated with several systemic diseases due to its ability to enter the bloodstream, especially during episodes of gum disease or tooth infections. Some of the systemic conditions include the following: cardiovascular disease since *S. mutans* has been implicated in the development of infective endocarditis, a life-threatening infection of the heart valves [44]; diabetes, because it can exacerbate insulin resistance and worsen the glycemic control in individuals with diabetes [45]; respiratory diseases, via aspiration pneumonia, where oral bacteria are inhaled into the lungs, associated with exacerbating chronic obstructive pulmonary disease (COPD), through the spread of bacteria to the lower respiratory tract [46]; Alzheimer’s disease and cognitive decline by triggering neuroinflammation, which is associated with cognitive decline and Alzheimer’s disease progression [47]; and preterm birth and pregnancy outcomes, by inducing inflammation [48].

*S. mutans* is among the most significant oral pathogens related to dental caries, although it may not be the most aggressive pathogen across all oral health conditions [49]. For example, *P. gingivalis* has been strongly associated with periodontal disease, and *C. albicans* can lead to oral thrush, especially in immunocompromised individuals [49]. Thus, the pathogenic impact of *S. mutans* is specific to its nutritional environment, it is particularly potent in caries development, while other pathogens may present greater risks in different conditions [39,42].

## 6. *P. gingivalis*

*P. gingivalis* is a bacterium associated with chronic periodontitis, a condition characterized by the inflammation and destruction of the gums, ligaments, and bone supporting the teeth [50]. The bacteria cause periodontitis, a localized inflammation in the periodontal ligament (gums), leading to the following symptoms: swelling, redness, bleeding, and receding gums. Over time, periodontitis can result in tooth mobility and, in severe cases, tooth loss [51]. *P. gingivalis* can stimulate an immune response that includes the activation of pro-inflammatory cytokines, which can lead to tissue destruction if left unchecked [52]. *P. gingivalis* also has mechanisms to evade immune detection, allowing it to persist in the periodontal tissues [53].

*P. gingivalis* has been implicated in several systemic diseases due to its ability to enter the bloodstream through inflamed gums. The diseases include cardiovascular disease, where the bacteria contribute to the development of arterial plaque, increasing the risk of atherosclerosis [54]. *P. gingivalis* is also thought to contribute to diabetes, by exacerbating insulin resistance and worsening the glycemic control in individuals with diabetes [55]. In rheumatoid arthritis, *P. gingivalis* triggers an autoimmune response that can worsen inflammation and joint damage [56]. In Alzheimer’s disease, the promotion of chronic inflammation might accelerate neurodegeneration [57]. The multiple pathogenic effects of *P. gingivalis* throughout the body have a significant effect on systemic health underscoring the importance of managing periodontal disease to protect overall well-being and reduce the risk of neurodegeneration in old age.

## 7. *F. nucleatum*

*F. nucleatum* is an abundant Gram-negative anaerobic bacillus bacterium commonly found in the oral cavity, where it plays a significant role in periodontal disease [58]. Known as a “bridging” bacterium, *F. nucleatum* connects early and late colonizers within dental biofilms, facilitating an anaerobic environment that supports the growth of other periodontal pathogens [59]. Beyond its effects in the oral cavity, *F. nucleatum* has systemic implications due to its ability to enter the bloodstream and colonize other body sites [60], contributing to disease beyond the mouth. *F. nucleatum* has been shown to enhance the proliferation of oral squamous cell carcinoma [61] and is implicated in DNA damage, tumor development, and metastasis in colorectal cancer [62]. *F. nucleatum* may promote carcinogenesis by stimulating abnormal cell growth and suppressing immune responses, by activating pathways that enhance cancer cell survival and metastasis [63]. 

*F. nucleatum* has been linked to adverse pregnancy outcomes, including preterm birth, low birth weight, and stillbirth [64]. It can reach the placenta or amniotic fluid, potentially through the bloodstream, where it induces inflammation that disrupts fetal development and contributes to the complications. 

Chronic inflammation caused by *F. nucleatum* in the oral cavity is associated with an increased risk of atherosclerosis and cardiovascular disease, particularly in individuals with periodontal disease [65]. *F. nucleatum* has also been implicated in gut dysbiosis and inflammation, which can contribute to gastrointestinal disorders such as inflammatory bowel disease, Crohn’s disease, and ulcerative colitis. Additionally, in combination with other oral pathogens, *F. nucleatum* has been implicated in neuroinflammatory conditions, such as Alzheimer’s disease [66] and infection-induced neurodegeneration [67], affecting brain health. *F. nucleatum* infections also produce succinic acid within tumors, which can reduce the efficacy of chemotherapy by promoting resistance in cancer cells [68]. The finding suggests that treating *F. nucleatum* infections with antibiotics could help re-sensitize tumors to immunotherapy, potentially enhancing the cancer treatment outcomes.

## 8. *A. actinomycetemcomitans*

*A. actinomycetemcomitans* (AA) is linked to aggressive periodontitis, characterized by rapid destruction of the periodontal tissues [69]. AA produces leukotoxin, lipopolysaccharide (LPS), other toxins, and pathogenic cytokines [70], which destroys leukocytes and contributes to systemic inflammation increasing the risk of other health complications [71]. The early stages of several systemic diseases are associated with high serum immunoglobulin (Ig)A and IgG antibody levels against AA, such as cardiovascular diseases [72], diabetes [73], rheumatoid arthritis [74], respiratory infections, adverse pregnancy outcomes, and pancreatic cancer [75]. The antimicrobial disinfection of periodontal pockets with mouthwash or laser therapy [76] may help alleviate the periodontal and systemic pathogenic effects of AA.

## 9. *P. intermedia*

*P. intermedia* is often found in periodontal pockets and is associated with chronic periodontitis and malignancy [77]. It is estimated that 20–40% of all fatal cancers in humans are caused by microorganisms, with the risk increasing in old age [78]. *P. intermedia* is found at elevated levels within colorectal adenocarcinoma (CRC) tissues and has been shown to enhance the migration and invasion capabilities of CRC cells [79]. Additionally, *P. intermedia* and *F. nucleatum* act synergistically to promote the malignant transformation of colorectal adenomas into carcinomas. In a rodent model, *P. intermedia* significantly promoted tumor growth, invasion, angiogenesis, and metastasis, markedly affected the levels of inflammatory cytokines, and markedly altered M2 macrophages and regulatory T cell (Treg) infiltration into the tumor microenvironment [78]. Thus, harboring *P. intermedia* and other oral pathogens has been linked to an increased risk of malignancy [77]. Investigations of the link between *P. intermedia* and malignancy could be instrumental in identifying patients at high risk of malignant progression or metastasis in CRC, allowing for more tailored and proactive clinical management [77].

## 10. *T. denticola*

*T. denticola* is a highly pathogenic spirochete bacterium that has been associated with periodontal disease and neuroinflammation, due to it residing in deep periodontal pockets around the teeth [80]. It is a principal member of the “red complex” of periodontal pathogens, along with *P. gingivalis* and *T. forsythia*. *T. denticola* plays a critical role in advancing periodontitis, gingivitis, gum recession, and potential tooth loss [81]. Another member of the genus Treponema, the subspecies, *T. pallidum*, is the causative agent of the sexually transmitted disease, syphilis, a chronic, multi-stage, human infection [82]. Recent evidence has detected *T. denticola* clusters in brain tissue sections of individuals with Alzheimer’s disease, suggesting a link between periodontal pathogens, neuroinflammation, and neurodegenerative disease processes [83].

*T. denticola* has been linked to gastrointestinal cancers, particularly in orodigestive and colorectal tissues, where it may evade immune surveillance, enabling abnormal cell proliferation [84]. Its pathogenic effects extend to cardiovascular disease, preterm low birth weight, diabetes mellitus, respiratory diseases, and osteoporosis [85]. Meanwhile, *T. pallidum*, the causative agent of syphilis, exhibits complex survival, invasion, and adaptation strategies that allow it to establish life-long infections, and it will invade all human tissue types if left untreated. Despite an incomplete understanding of *T. pallidum* pathogenesis, syphilis remains highly treatable with antibiotics, due in part to the pathogen’s relatively short incubation period, making eradication a plausible goal [82].

## 11. *T. forsythia*

*T. forsythia* is a key bacterium associated with periodontal disease, where it contributes to tissue destruction and inflammation in the gums [86]. Through its virulence factors, *T. forsythia* promotes immune evasion and enhances inflammatory responses, accelerating the breakdown of bleeding gum tissue and bone loss, which are hallmarks of chronic periodontal disease [87]. *T. forsythia*’s role in dysbiosis, i.e., imbalance of the oral microbiome, can further exacerbate the severity of periodontal disease, not only in adults, but also in offspring, likely through vertical saliva transmission [88]. Additionally, recent studies suggest that periodontal pathogens like *T. forsythia* may also impact systemic health, exacerbating conditions such as cardiovascular disease, diabetes, and even adverse pregnancy outcomes by promoting inflammation and releasing toxins that can circulate throughout the body [89]. The presence of *T. forsythia* in the oral cavity has been linked to an increased risk of fever in elderly nursing home residents [90], suggesting a potential role in triggering systemic inflammatory responses. Patients with elevated levels of periodontal pathogens in their saliva also tend to experience a higher incidence of chronic diseases, including multimorbidity, tissue and organ function loss, and autoimmune conditions such as diabetes mellitus, cardiovascular disease, rheumatoid arthritis, systemic lupus erythematosus, psoriasis, cancer, and neuropsychiatric disorders [91]. The association underscores the impact of oral pathogen burden on broader health challenges, particularly in vulnerable populations, emphasizing the need for new therapeutic targets to manage the extensive systemic consequences of oral infections.

## 12. Diseases

**Figure 4:**
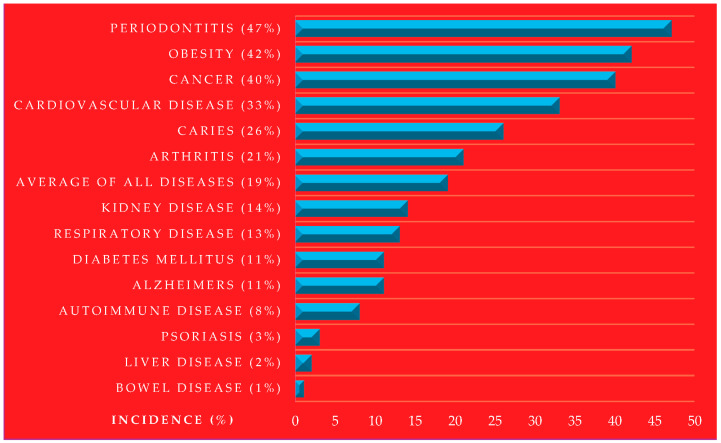
Bar chart of the incidences of diseases. The bar chart shows the incidence of diseases among Americans from the published literature, including data gathered by the Centers for Disease Control. Commonly, people can suffer from multiple diseases at the same time, especially with advancing age. Some of the incidence rates are controversial, because it is dependent on the method of analysis and calculation; the incidence rates here are generally within the medium range of the average for each disease.

Ninety percent of the United States USD 4.5 trillion in annual healthcare expenditures are due to chronic and mental health conditions [92,93]. Heart disease and stroke are the leading causes of death for one-third of American adults, with one million deaths each year [94]. In the United States, 1.7 million people are diagnosed with cancer each year, and over 600,000 lose their lives to the disease, making it the nation’s second-leading cause of death [95,96]. Over 38 million Americans live with diabetes, while an additional 98 million adults in the U.S. have prediabetes, putting them at high risk of developing type 2 diabetes [97]. Diabetes can lead to severe complications, including heart disease, kidney failure, and blindness [98]. The most significant diseases according to the estimated proportions of American adult sufferers are the following: periodontal disease (47.2%) [99], Obesity (42.4%) [100], Cancer (40.5%) [101], Cardiovascular disease (33%) [102], untreated dental caries (26%) [103], arthritis (21.2%) [104], kidney diseases (14%) [105], respiratory (13.2%) (asthma 8.9% and chronic obstructive pulmonary disease 4.3%) [106], diabetes (11.3%) [107], Alzheimer’s disease in Americans aged over 65 years (10.9%) [108], autoimmune disease (8%) [109], psoriasis (3%) [110] liver disease (1.8%) [111], and bowel disease (1%) [112]. These are shown in Figure 4.

**Figure 5:**
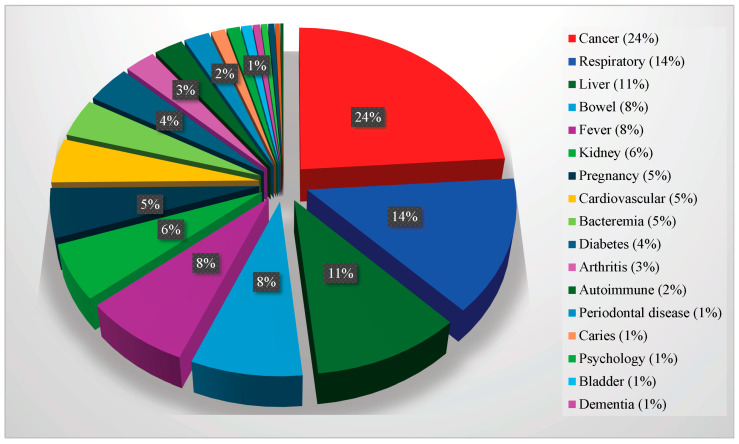
Pie chart of oral pathogen disease research. The pie chart shows the proportion of total publications available by bacteria (including oral pathogens) and disease, from a PubMed search conducted on 14 November 2024, with no time limit.

The most significant diseases associated with bacteria (oral pathogens) according to the proportions of publications in PubMed are the following: cancer (24%), respiratory (14%), liver (11%), bowel (8%), fever (8%), kidney (6%), pregnancy (5%), cardiovascular (5%), bacteremia (4%), diabetes (4%), arthritis (3%), autoimmune (2%), periodontal disease (1%), caries (1%), psychological (1%), bladder (1%), dementia (1%), lupus (1%), Alzheimer’s (1%), arteriosclerosis (1%), and psoriasis (0%). These are shown in Figure 5.

The comparison of the disease burden in Figure 5 with the proportion of articles published in Figure 4 highlights a stark disparity: research on oral pathogens, periodontitis, dental caries, and several other diseases remains disproportionately under-researched and under-published (1% of articles) relative to their substantial health burden (affecting 26–47% of Americans). The disparity likely stems from negative research bias, and the inadequate funding for oral pathogen research. Addressing the imbalance through increased research investment could yield significant benefits, including improved health outcomes, enhanced quality of life, and reduced economic burdens associated with disability and chronic disease management.

## 13. Periodontitis

Periodontal diseases arise from an imbalance, or dysbiosis, within the oral microbiota, triggering host immune responses through the pathways linked to proatherogenic processes [113]. Periodontal disease impacts 47.2% of American adults aged 30 and older, with its prevalence rising significantly with increased age; around 70% of adults aged 65 and older are affected [99]. Oral pathogens and microbiota dynamically interact with the host, through infection, immunity, and metabolism, with the systemic organs [114]. Oral pathogens and salivary microbiota serve as biomarkers of oral health and systemic disease [115]. Oral pathogens are connected to systemic health via three metastatic pathways, namely transient bacteremia leading to infection, immuno-logical injury, and toxic injury [116], through alterations in serum lipid levels [117]. 

The pathways contribute to a range of divergent diseases, including acute bacterial myocarditis, infective endocarditis, brain abscesses, uveitis, iridocyclitis, trigeminal and atypical facial neuralgia, unilateral facial paralysis, fever of “unknown” origin, and neutrophil dysfunction [116]. 

Periodontitis and cardiovascular diseases (CVDs) are chronic, progressive conditions that often begin in adolescence. Sufferers of periodontitis face a 24–34% increased risk of coronary artery disease (CAD) [118], ischemic stroke [119], and atherosclerotic vascular disease [120], primarily caused by the interplay between cholesterol buildup, infection, and the inflammation adaptive responses of T and B cells [121]. Periodontal diseases are also associated with an increased risk of developing autoimmune diseases and mental health issues [122], diabetes, psoriasis, rheumatoid arthritis, pregnancy outcomes and respiratory diseases [123], cognitive neuro-degenerative, and autoimmune diseases [124] which creates a substantial public health burden [122]. 

## 14. Dental Caries

**Figure 6:**
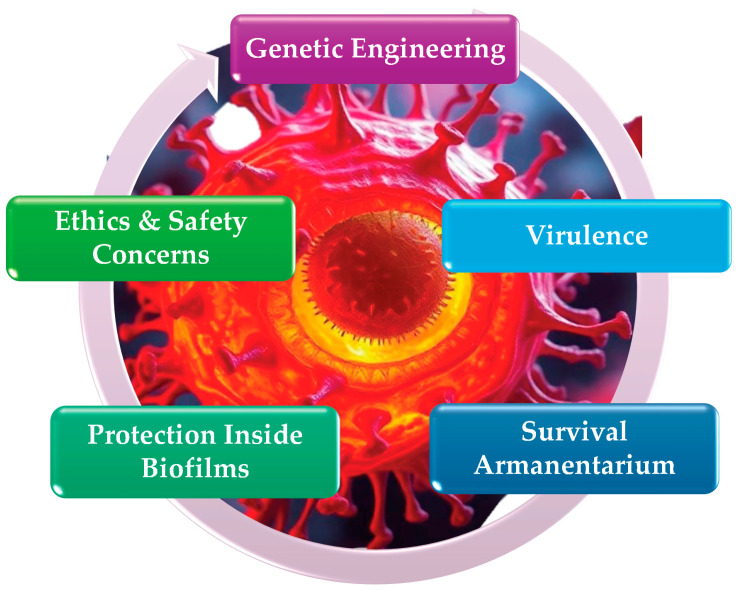
Vaccine for oral pathogens. The oral pathogen vaccines, which have been under development for decades, face significant ethical and safety concerns due to pathogen virulence, genetic engineering variability, survival armamentarium, and protection within biofilms. A primary challenge is the potential risk of inadvertently bioengineering pathogens with heightened resistance to antibiotics, raising concerns about creating “superbugs” that could exacerbate public health issues.

Oral pathogens, primarily *S. mutans*, Lactobacillus, and Actinomyces species, metabolize sugars from the host’s diet, producing acids that dissolve tooth enamel and dentin, leading to dental caries or tooth decay [25]. Dental caries create a significant health burden, affecting approximately 90% of American adults. Among adults aged 20–64, around 26% currently have untreated tooth decay. The prevalence among children is also concerning, with approximately 52% of those aged 6–8 having experienced cavities in their primary teeth and nearly 57% of adolescents aged 12–19 having cavities in their permanent teeth [103]. Over time, repeated acid attacks weaken the enamel, eventually forming a cavity or hole in the tooth. If left untreated, the decay can progress into the deeper layers of the tooth, causing pain, and infection, ultimately leading to tooth loss. A successful caries vaccine that inhibits the activity of *S. mutans* has eluded researchers for decades, due to its virulence, genetic engineering variability, survival armamentarium, protection within biofilms [125], ethics and the safety concerns regarding bioengineering super-antibiotic-resistant pathogens, as shown in Figure 6.

## 15. Cancer

Women who suffer from periodontitis are up to three times more likely to develop breast cancer [126]. Approximately 40.5% of Americans will be diagnosed with cancer at some point in their lives, encompassing all types of cancer [101]. The risk varies depending on factors such as age, lifestyle, genetics, and environmental exposures [101]. Cancer continues to be one of the leading causes of death in the United States, highlighting its significant impact on public health.

Growing evidence from epidemiological and genetic studies suggests a significant link between oral pathogens and certain cancers, including oral, digestive tract, esophageal, pancreatic, and colorectal cancers. The chronic inflammation caused by oral pathogens plays a critical role in the development and progression of these malignancies.

For instance, *P. gingivalis* has been detected in 61% of esophageal cancer tissues, compared to 12% in adjacent non-cancerous tissues and 0% in normal esophageal mucosa [127]. Similarly, periodontal disease pathogens have also been implicated in colorectal cancer by inducing excessive immune responses and activating cancer growth genes [128,129,130]. The bacteria preferentially accumulate in adenomas—benign bowel growths that can progress to cancer—and thrive in the nutrient-rich microenvironment of colonic lesions due to their asaccharolytic metabolism [131].

The key periodontal pathogens, *P. gingivalis* and *F. nucleatum*, are pivotal in promoting carcinogenesis [132]. They sustain a chronic inflammatory state in the local environment and protect carcinomas from immune detection and attack [133], thereby facilitating tumor progression and survival.

*Fusobacteria* also invade the bowel by attracting specific immune cells, triggering inflammatory responses that may accelerate colorectal tumor formation. The bacterium’s unique surface molecules enable the bacteria to attach to and invade colorectal cancer cells. In colorectal cancer, *F. nucleatum* has been shown to expand myeloid-derived immune cells, inhibit T-cell activation, and induce T-cell apoptosis [133]. Furthermore, *F. nucleatum* shields tumor cells from immune detection and destruction. Its Fap2 protein directly interacts with TIGIT (T-cell immunoglobulin and ITIM domain), suppressing NK cell cytotoxicity and impairing T-cell responses [75]. Oral pathogens, particularly *P. gingivalis* and *A. actinomycetemcomitans*, have been associated with an increased risk of pancreatic cancer [134]. The mechanisms via which the pathogens contribute to carcinogenesis include triggering excessive inflammatory responses, suppressing the host immune system, promoting the malignant transformation of cells, inhibiting apoptosis, and secreting carcinogenic substances [134] that induce genetic mutations in host cells. The salivary microbiota, comprising both oral pathogens and commensal microorganisms, have been associated with the development of digestive tract cancers [135]. The connection suggests the potential involvement of unidentified viruses, bacteria, and environmental factors as additional carcinogenic contributors.

## 16. Respiratory Diseases

People with periodontitis are 2.1 times more likely to suffer from chronic obstructive pulmonary disease (COPD) [136]. Respiratory diseases remain a major public health issue in the U.S., significantly impacting individuals’ quality of life and placing a burden on healthcare systems. Asthma affects 25 million Americans, while over 15 million adults have been diagnosed with chronic obstructive pulmonary disease (COPD) [106]. The figures represent only a portion of the broader spectrum of respiratory diseases, which also include conditions like pneumonia, pulmonary fibrosis, and other chronic lung diseases. 

Oral pathogens have been implicated in the development and exacerbation of respiratory diseases, primarily through mechanisms such as aspiration or the direct colonization of the respiratory tract. Pathogens from the oral cavity can be aspirated into the lower airways, trachea, and lungs, where they may be expelled through ciliary actions or coughing [137]. However, due to the lack of mucosal environments conducive to bacterial colonization, the microbial density in the lungs is typically much lower—about 1/1000th of that found in the oral cavity [137,138]. Despite their physiology, oral pathogens are still associated with chronic respiratory conditions due to their proximity to the lungs [138]. The aspiration of the pathogens can lead to dysbiosis in the lung microbiota, triggering inflammatory responses marked by elevated neutrophils and lymphocytes [139].

A randomized controlled trial found that asthmatic patients had a bronchial microbiome enriched with periodontal pathogens like *Fusobacterium* and *Porphyromonas* [140,141,142,143]. In addition, elevated levels of inflammatory cytokines, including TNF-α [143], VCAM-1, ICAM-1, RAGE [143], CRP, and IL-6 [144], have been observed in individuals with periodontitis and chronic respiratory diseases like COPD. Oral pathogens such *as P. gingivalis*, *F. nucleatum*, and *A. actinomycetemcomitans* can be aspirated into the lungs, leading to infections such as aspiration pneumonia [145]. 

The respiratory and gut microbiota play a critical role in protecting against pneumonia by preventing the colonization of pathogenic bacteria and modulating the immune responses. Dysbiosis in the respiratory microbiota has been considered a significant risk factor for pneumonia, with certain microbiota profiles dominated by *Lactobacilli*, *Rothia*, and *Streptococcus* being strongly associated with pneumonia development [146]. Additionally, specific levels of IgA production against pathogens like *Lactobacillus*, *Prevotella*, *Veillonella*, *Bacteroides*, and *Streptococcus* are linked to reduced pneumonia risk [147]. *P. gingivalis* has been detected in the bronchoalveolar lavage fluid of emphysema patients, particularly those who have undergone lung transplantation [148]. Elevated IgA antibodies against *F. nucleatum* were found to be 3.5 times higher in bronchitis patients compared to healthy individuals [149].

Patients with periodontal and gingival disease and virulent oral pathogens tend to experience more severe respiratory symptoms and complications when infected by severe acute respiratory syndrome (SARS) and COVID-19 [150]. COVID-19 likely has severe long-haul pathogenic effects on periodontal, gingival, respiratory, cardio-vascular, cerebrovascular, neurologic, enteritis conditions and diseases. The chronic inflammation associated with periodontal disease can exacerbate the inflammatory response to these viral infections, resulting in worse outcomes, more severe symptoms, a higher risk of ICU admission, the need for assisted ventilation, and increased mortality [151].

## 17. Liver Diseases

People with severe periodontitis are more than two times more likely to suffer from cirrhosis, or end-stage chronic liver disease [152,153]. Individuals with periodontitis are 2.7 times more likely to suffer inflammatory bowel disease (IBD), 2.2 times more likely to suffer Crohn’s disease, and 3.5 times more likely to suffer from ulcerative colitis [154].

Chronic liver disease, including cirrhosis, affects approximately 4.5 million Americans [111] and is primarily caused by factors such as viral infections (particularly Hepatitis C), excessive alcohol consumption, obesity, and non-alcoholic fatty liver disease (NAFLD). The conditions contribute to liver damage, inflammation, and scarring that can ultimately lead to liver failure and other serious health complications. NAFLD is estimated to affect about 25% of the U.S. population, with a higher prevalence in individuals who are obese or have diabetes [155]. 

The oral pathogen, *P. gingivalis*, may indirectly contribute to the development or exacerbation of NAFLD. *P. gingivalis* is an inducer of chronic inflammation in the oral cavity, and its bacterial products, such as lipopolysaccharides (LPS) [156], as well as pro-inflammatory cytokines like TNF-α, IL-6, and CRP [157], can enter the bloodstream through inflamed gum tissue. Once the products enter the bloodstream, they can accumulate in the liver, contributing to the development or worsening of NAFLD [158].

Additionally, *P. gingivalis* may directly infect and colonize the liver, triggering chronic inflammation and fibrosis that can progress to more severe stages of liver disease, including non-alcoholic steatohepatitis (NASH) and cirrhosis [156,157,158]. The chronic systemic inflammation induced by *P. gingivalis* may also exacerbate insulin resistance, a key factor in the progression of NAFLD. Furthermore, *P. gingivalis* and its metabolic products can increase the oxidative stress in the liver, contributing to cell damage and liver injury. The oxidative stress is a significant driver of NAFLD progression, leading to fat accumulation, liver cell damage, and fibrosis, and eventually leads to liver cirrhosis and hepatocellular carcinoma [158].

## 18. Bowel Diseases

People with periodontitis are 2.7 times more likely to suffer inflammatory bowel disease (IBD), 2.2 times more likely to suffer Crohn’s disease, and 3.5 times more likely to suffer from ulcerative colitis [159].

The oral pathogens, *P. gingivalis*, *F. nucleatum*, and others have been strongly implicated in the development and exacerbation of various gastrointestinal disorders [159], particularly IBD [160], colorectal cancer (CRC) [161], ulcerative colitis [162], and Crohn’s disease [162]. While these conditions affect less than 1% of Americans, they predominantly impact adults and contribute significantly to morbidity [112]. 

Oral pathogens can translocate from the oral cavity to the gastrointestinal tract via aspiration or other pathways. Once there, they colonize the intestines, disrupt the gut microbiome, and alter immune responses [159]. Dysbiosis, or microbial imbalance, in the gut is a well-established factor in IBD and other gastrointestinal disorders [162]. The pathogens also release byproducts such as lipopolysaccharides (LPS) and pro-inflammatory cytokines that can enter the bloodstream and trigger systemic inflammation exacerbating intestinal conditions like Crohn’s disease and ulcerative colitis [162]. Furthermore, oral pathogens modulate immune responses, inducing immune tolerance or chronic inflammation that can increase the susceptibility to IBD and facilitate cancer progression by helping malignant cells evade immune detection [163].

*F. nucleatum* is linked to chronic inflammation and colorectal cancer, because it promotes tumor growth by interacting with immune cells in the colon, driving inflammatory processes, and interfering with tumor suppressor genes [161]. Specifically, *F. nucleatum* uses surface proteins like Fap2 to interact with cancer cells, to suppress immune responses, and to enhance tumor proliferation [161].

Additionally, oral bacteria produce toxic metabolic byproducts that can damage the gut lining, leading to conditions like leaky gut syndrome [164]. The damage allows harmful substances to enter the bloodstream, further triggering inflammation and immune activation in distant organs, including the intestines.

Overall, oral pathogens contribute to bowel diseases through the direct colonization of the gastrointestinal tract, the induction of chronic inflammation, the disruption of immune responses, and the promotion of precancerous gastric lesions [165]. For example, chronic periodontal disease and colonization by periodontal pathogens have been linked to an increased risk of precancerous gastric lesions [166], emphasizing the systemic implications of oral health for gastrointestinal conditions.

## 19. Fever and Bacteremia

Periodontal root scaling treatments can increase a patient’s risk of suffering bacteremia (the presence of bacteria in the bloodstream) by up to 75% [167]. The entry of periodontal oral pathogens into the bloodstream may cause a persistent fever [168]. 

Oral pathogens can play a significant role in the development of fever and bacteremia, particularly in individuals with periodontal disease [169] or other oral infections. Oral infections, such as periodontitis or dental abscesses, enable pathogens to enter the bloodstream through inflamed or damaged gingival tissues, along with bacterial toxins and byproducts. The process stimulates systemic inflammation, often manifesting as a fever [168]. The body’s immune system detects bacterial components, such as lipopolysaccharides (LPS) from Gram-negative pathogens like *P. gingivalis* or peptidoglycans from Gram-positive pathogens like *S. mutans*. In response, the immune system releases pyrogens (fever-inducing substances) such as cytokines (IL-1, IL-6, and TNF-α) that signal the hypothalamus to raise the body’s temperature, resulting in a fever [170].

In healthy individuals, transient bacteremia is typically cleared by the immune system. However, in those with a weakened immunity or underlying conditions, bacteremia can progress to severe complications, including sepsis, endocarditis, or the colonization of distant tissues [171]. Persistent bacteremia may lead to systemic complications such as organ abscesses, joint infections, or metastatic infections like brain abscesses [172]. Patients at high risk, such as those with prosthetic heart valves or a history of endocarditis, are often prescribed prophylactic antibiotics before invasive dental procedures to minimize the risk of bacteremia and its complications [173]. The systemic impact of oral pathogens highlights the critical importance of maintaining good oral hygiene and addressing periodontal infections promptly. Doing so reduces the risk of bacteremia, fever, and severe systemic conditions.

## 20. Kidney and Bladder Disease

People with periodontitis are 3.8 times more likely to suffer from chronic kidney disease (CKD) [174]. The antibodies to the oral pathogens, *T. forsythia* and *T. denticola*, inversely predict the risk of bladder cancer by 1.7 times and by 1.6 times [175]. Oral pathogens, particularly those associated with periodontal disease, have been implicated in the development and progression of chronic kidney disease (CKD) [176]. CKD, which affects millions worldwide, is characterized by a gradual decline in kidney function, caused by an irreversible reduction in functional nephrons [177]. Emerging evidence suggests that systemic inflammation and infections play a critical role in its pathogenesis. Approximately 37 million Americans, that is, one in seven adults, are affected by CKD [105].

Periodontal pathogens such as *P. gingivalis*, *F. nucleatum*, and *A. actinomycetem-comitans* can enter the bloodstream through inflamed and bleeding gums. Once in the circulation, these bacteria and their byproducts—such as lipopolysaccharides (LPS) and pro-inflammatory cytokines (e.g., TNF-α, IL-6, and CRP), and oxidative stress—promote systemic inflammation [177]. Systemic inflammation is a recognized risk factor for kidney damage and accelerates CKD progression by inducing endothelial dysfunction, oxidative stress, and increased vascular permeability, all of which can compromise kidney function over time [176].

The inflammatory mediators associated with oral pathogens contribute to endothelial dysfunction, impairing the blood flow and filtration processes vital to kidney health. Periodontitis-associated inflammation has been linked to microvascular damage, which hastens the decline in the glomerular filtration rate (GFR)—a key marker of CKD severity [177]. The chronic exposure to oral pathogens can also result in immune dysregulation, where the immune system becomes overactive or misdirected. In CKD patients, this dysregulation exacerbates kidney tissue damage, potentially accelerating disease progression. Studies have shown that individuals with severe periodontal disease are at a significantly higher risk of developing end-stage renal disease [178]. The relationship between CKD and periodontal disease is bidirectional in the form of a feedback axis because poor oral health worsens kidney outcomes, while kidney dysfunction weakens the immune defenses, increasing the susceptibility to oral infections [178].

Interventions such as periodontal treatments have been shown to reduce systemic inflammation and improve the markers of kidney function in CKD patients [179]. By addressing chronic oral infections, the inflammatory burden on the kidneys can be mitigated, potentially slowing the progression of CKD [180]. The strong association between oral pathogens, kidney disease, and bladder disease underscores the importance of maintaining good oral health as a preventive strategy for systemic conditions, including CKD. Further research is needed to fully elucidate the mechanisms underlying this relationship and to develop integrated approaches for managing both oral and renal health effectively.

## 21. Pregnancy Complications

Pregnant women with severe periodontal disease are 7.5 times more likely to go into labor prematurely [181]. Oral pathogens have been strongly associated with various pregnancy complications, including preterm birth (before 37 weeks of gestation) [182], and low birth weight [183], emphasizing the vital role of maternal oral health in ensuring favorable pregnancy outcomes. The abundance of the oral pathogens, *P. gingivalis*, *A. actinomycetemcomitans*, and *C. albicans*, significantly increases during early pregnancy [184]. The increase is likely influenced by physiological changes and elevated levels of female sex hormones, which can alter the oral microbiome and promote the growth of pathogenic microorganisms [185].

Periodontal disease, a chronic oral infection caused by pathogens such as *P. gingivalis*, *F. nucleatum*, and *A. actinomycetemcomitans*, contributes to systemic inflammation that can adversely affect both the mother and the developing fetus, with the risk of preterm delivery (before 37 weeks of gestation) and delivering babies with a low birth weight. The systemic inflammation triggered by oral pathogens stimulates the release of cytokines like IL-6, TNF-α, and prostaglandins, which can promote uterine contractions and early labor. Maternal periodontitis may affect the offspring’s epigenome and result in some health consequences in adult life. [186].

Preeclampsia, a serious condition characterized by high blood pressure and damage to organs such as the liver or kidneys, has been linked to maternal periodontal disease [187]. Oral pathogens and their inflammatory byproducts can migrate into the bloodstream, contributing to endothelial dysfunction and elevated vascular inflammation—key factors in the development of preeclampsia [188]. Oral pathogens such as *S. mutans*, which is associated with dental caries, can be transmitted from mother to child during pregnancy or postpartum [189]. The transmission increases the risk of early childhood caries, emphasizing the importance of maternal oral health. 

The oral pathogen, *F. nucleatum*, has been detected in cases of intra-amniotic infections, neonatal sepsis, and hypertensive disorders of pregnancy, particularly in instances of preterm birth and fetal loss [64]. *F. nucleatum* is thought to be translocated from the oral cavity to the uterine environment via the bloodstream, where it disrupts placental function and triggers inflammatory responses that can harm the fetus [64]. Periodontal disease has been linked to an increased risk of gestational diabetes mellitus, potentially due to systemic inflammation exacerbating insulin resistance [190].

Pregnant women are encouraged to practice excellent oral hygiene, including regular brushing, flossing, and professional dental cleanings. By addressing oral infections promptly and maintaining good oral health, the risk of the pregnancy complications associated with oral pathogens can be significantly reduced [191], improving the outcomes for both mother and child.

## 22. Cardiovascular Disease and Arteriosclerosis

People with periodontitis are more than twice as likely to suffer a stroke [192] and are 3 to 5.7 times more likely to suffer heart failure [193]. Oral pathogens, particularly those associated with periodontal disease—such as *P. gingivalis*, *A actinomycetemcomitans*, *F nucleatum*, and *S. mutans* (commonly linked to dental caries)—have been strongly implicated in the development and progression of cardiovascular diseases (CVDs) [194]. These conditions include atherosclerosis (arterial thickening and hardening) [195], myocardial infarction (heart attack) [196], stroke, thrombosis (blood clots) [197], hypertension (increased blood pressure) [193], and infective endocarditis (infection of the heart valves) [198]. CVDs and arteriosclerosis affect up to 33% of Americans and remain the leading causes of mortality [102]. The systemic impact of oral pathogens on cardiovascular health is becoming increasingly clear, underscoring the critical connection between oral health and overall well-being.

Oral pathogens and their byproducts, such as lipopolysaccharides (LPSs) and pro-inflammatory cytokines, can enter the bloodstream through bleeding gums, triggering systemic inflammation and contributing to endothelial dysfunction in cardiovascular tissues [199]. This dysfunction impairs the ability of the blood vessels to regulate blood flow and repair damage, promoting the development of atherosclerosis—the accumulation of plaques on arterial walls. Studies have shown that individuals with periodontal disease have a 20–25% higher risk of developing CVDs [200]. Additionally, the presence of Cnm-positive *S. mutans* has been linked to cerebral microbleeds [201], further illustrating the connection between oral health and systemic vascular conditions [200,201].

Oral pathogens can invade endothelial and phagocytic cells within the atheroma, causing pathogenic changes and a progression of lesions. The oral pathogens release inflammatory mediators such as C-reactive protein (CRP), fibrinogen, and metalloproteinases from periodontal lesions into the systemic circulation [202]. The oral pathogens play a significant role in the development and progression of CVDs by triggering systemic inflammation, impairing vascular function, and promoting atherosclerosis and thrombosis. The connection underscores the importance of maintaining good oral hygiene and promptly treating periodontal infections [203] as a preventive measure for cardiovascular health.

## 23. Diabetes Mellitus

Individuals with severe periodontal disease caused by oral pathogens are up to two to three times more likely to develop type 2 diabetes mellitus (T2DM) [204]. The relationship between periodontal disease and diabetes is a bidirectional feedback axis, with each condition worsening the other. Periodontal infections trigger systemic inflammation and insulin resistance, leading to poor glycemic control, while diabetes exacerbates periodontal disease by impairing the immune function and amplifying inflammatory responses [205]. The interconnected dynamic is driven by chronic inflammation, immune dysfunction, and metabolic dysregulation [205].

Diabetes mellitus comprises a group of metabolic disorders characterized by hyperglycemia, arising from impaired carbohydrate metabolism due to glucose underutilization and overproduction [206]. Type 1 diabetes mellitus is an autoimmune condition resulting in the destruction of pancreatic islet β-cells, caused by insulin resistance coupled with inadequate insulin secretion [206]. Gestational diabetes mellitus, which occurs in about 17% of pregnancies, resolves in postpartum but significantly increases the risk of future T2DM [107].

Oral periodontal pathogens such as *P. gingivalis* and *F. nucleatum* release lipopolysaccharides (LPSs) that enter the bloodstream through inflamed gums and translocate over oral mucosa [207]. LPSs promote the release of pro-inflammatory cytokines such as tumor necrosis factor-alpha (TNF-α), interleukin-6 (IL-6), and C-reactive protein (CRP) [208]. The mediators impair insulin signaling, increase insulin resistance, and exacerbate hyperglycemia [209]. Persistent periodontal infections are linked to elevated hemoglobin A1c (HbA1c) levels, a marker of long-term glycemic control [210]. Untreated periodontal disease contributes to poor glucose regulation in diabetic patients [211]. Hyperglycemia in saliva and gingival crevicular fluid can create an environment conducive to pathogenic bacterial growth, accelerating periodontal disease progression [212]. 

Diabetes impairs the immune function, reduces neutrophil activity and phagocytosis, and enables oral pathogens like *P. gingivalis* and *A. actinomycetemcomitans* to proliferate [213]. The reduced blood flow and oxygen delivery in diabetic patients impair wound healing, complicating the resolution of oral infections [214]. Diabetic patients experience more severe periodontal tissue destruction and bone loss due to heightened immune and inflammatory responses to oral pathogens [215]. Oral pathogens exacerbate diabetes through systemic inflammation, impaired insulin sensitivity, and direct effects on glycemic control. Conversely, diabetes worsens oral infections by creating a favorable environment for pathogenic bacteria and impairing immune responses. The bidirectional feedback axis relationship between oral pathogens and diabetes [215] underscores the importance of integrated management. Addressing periodontal infections through timely treatment and maintaining optimal glycemic control can significantly reduce the burden of both conditions, preventing systemic complications and improving overall health outcomes.

## 24. Arthritis, Psoriasis, Autoimmune, and Chronic Inflammatory Diseases

*P. gingivalis* oral infections exacerbate the development and severity of collagen-induced rheumatoid arthritis (RA) [216,217]. Conversely, RA significantly increases the likelihood of edentulism (total tooth loss) by 4.5 times and raises the risk of periodontitis by 1.8 times [218]. Patients with RA also exhibit a higher prevalence of viral infections, including Epstein–Barr virus, varicella-zoster virus, human parvovirus B19, and herpes simplex virus type II [219,220]. RA is a chronic autoimmune disease affecting approximately 20% of U.S. adults aged 18 and older and encompasses nearly 100 conditions targeting joints and the surrounding tissues [104]. The inheritance, estimated at 60–65%, underscores a strong genetic predisposition [221]. Notably, the incidence and prevalence of autoimmune diseases are rising by 12–19% annually, likely driven by environmental factors triggering autoimmune inflammatory reactions [222].

Psoriasis is associated with the distinct characteristics of the salivary microbiota and altered salivary levels of inflammation-related proteins, differing significantly from those observed in patients with periodontitis or orally healthy individuals [223]. The administration of oral probiotics to target and reduce oral pathogens may provide health benefits for individuals with psoriasis by modulating the oral microbiota, reducing systemic inflammation, and potentially alleviating disease symptoms [224].

Immune disturbances in preclinical RA often originate outside the joints, particularly at the mucosal surfaces such as the periodontal tissues, lungs, or gut [225]. Oral pathogens like *P. gingivalis*, *P. intermedia*, *F. nucleatum*, and *A. actinomycetemcomitans* are primary contributors, capable of entering the bloodstream, disrupting immune responses, and sustaining chronic inflammation. The processes establish a critical link between poor oral health and systemic conditions like RA [226]. Persistent oral infections, including periodontitis and dental caries, significantly contribute to autoimmune chronic inflammatory diseases such as RA [227]. However, reducing the oral pathogen burden through non-surgical periodontal treatment can markedly alleviate RA symptoms within three months [228].

## 25. Alzheimer’s, Dementia, and Mental Health

Oral pathogens have been detected in the brains of individuals with Alzheimer’s disease (AD) [229]. Severe periodontal disease increases the risk of developing AD by 4.9 times and mild cognitive impairment by 2.3 times [230]. Alzheimer’s disease is a complex neurodegenerative disorder and the leading cause of dementia, characterized by progressive memory loss, cognitive decline, and mood alterations. The hallmark pathological features of AD include amyloid-beta (Aβ) plaques and neurofibrillary tangles (NFTs), which serve as key diagnostic markers, typically confirmed at autopsy [231]. In addition to Aβ plaques and NFTs, other neuropathological changes in AD include neuronal and synaptic loss, glial cell activation, and breaches in the blood–brain barrier, all of which contribute to its progression and serve as indicators of the underlying pathophysiological processes [232].

Infection by *P. gingivalis* and its lipopolysaccharides (LPSs) activates the complement cascade, increasing Aβ production and triggering the release of pro-inflammatory cytokines. The cascade promotes age-related brain inflammation, neuroinflammation, and neurodegeneration [233]. Oral pathogens also induce systemic antibody responses and hyperactivation of the hypothalamic–pituitary–adrenal (HPA) axis, further accelerating the progression of Alzheimer’s disease, dementia, and the associated mental health conditions like depression [234].

## 26. Systemic Lupus Erythematosus

Individuals with systemic lupus erythematosus (SLE) are up to 5.3 times more likely to be suffering from oral pathogens severely infecting and inflaming their periodontal tissues, causing periodontitis [235]. Oral periodontal pathogens can exacerbate SLE symptoms by increasing systemic inflammation, potentially triggering flares in autoimmune activity [236]. The composition of the oral pathogen microbiota in SLE is altered, independent of periodontal status [237]. Both periodontitis and SLE share overlapping inflammatory pathways [238], including the increased expression of both TLR-2 and TLR-4 receptors that trigger autoimmune reactions [239] and the dysregulation of the Th1 and Th2 cells of the adaptive immune system [240]. The bidirectional feedback axis relationship [241] consists of oral pathogens influencing systemic health, and *vice versa* [242].

Periodontal pathogens initiate inflammation and immune responses that, in the context of SLE, can further disrupt the immune regulation, leading the body to attack its own tissues [243]. Elevated levels of pro-inflammatory cytokines, such as TNF-α, IL-6, and IL-17, are central drivers of tissue destruction and disease progression in both conditions [240]. Additionally, SLE patients frequently experience oral manifestations such as ulcers, a dry mouth (xerostomia), and gingival inflammation, all of which predispose an individual to periodontal disease [244]. The SLE-related oral changes weaken the immune system’s ability to combat periodontal infections, exacerbating gum disease. Maintaining good oral health and preventing periodontitis may offer a cost-effective strategy to mitigate the progression of SLE [235]. Individuals with SLE face a higher risk of severe periodontitis due to a combination of impaired immune function and the effects of immunosuppressive medications, which further compromise oral health [237]. Treating periodontal disease through targeted therapies and the elimination of oral pathogens could help reduce systemic inflammation, alleviate SLE symptoms, and significantly improve the overall quality of life for those with SLE.

## 27. Obesity

Periodontitis, driven by pathogenic bacteria like *P. gingivalis* and *T. denticola*, induces chronic inflammation in periodontal tissues [50,80]. The pathogens release virulence factors such as lipopolysaccharides (LPSs) that activate immune cells and trigger the release of pro-inflammatory cytokines, including tumor necrosis factor-alpha (TNF-α), interleukin-6 (IL-6), and interleukin-17 (IL-17) [142,143,144]. The cytokines not only contribute to periodontal tissue destruction but can also exacerbate the systemic inflammatory conditions.

Oral pathogens contribute to the changes in the oral and gut microbiota through swallowed bacteria or inflammatory mediators [159]. This can lead to dysbiosis in the gut microbiome, and dysbiosis associated with oral infections [162] that may lead to energy metabolism and fat storage, exacerbating obesity [245]. Furthermore, systemic inflammation driven by oral pathogens can lead to adipocyte dysfunction, disrupting lipid metabolism and promoting the secretion of leptin and other adipokines that exacerbate energy imbalance and fat accumulation [246]. Addressing oral pathogens through enhanced oral hygiene, targeted periodontal therapies, or the administration of probiotics may help mitigate systemic inflammation and improve metabolic health [24]. The interventions highlight the critical role of oral health in managing chronic systemic conditions like obesity [245], underscoring the interconnectedness of oral and systemic well-being.

## 28. Oral Pathogens and an Increased Incidence of Systemic Diseases

**Figure 7:**
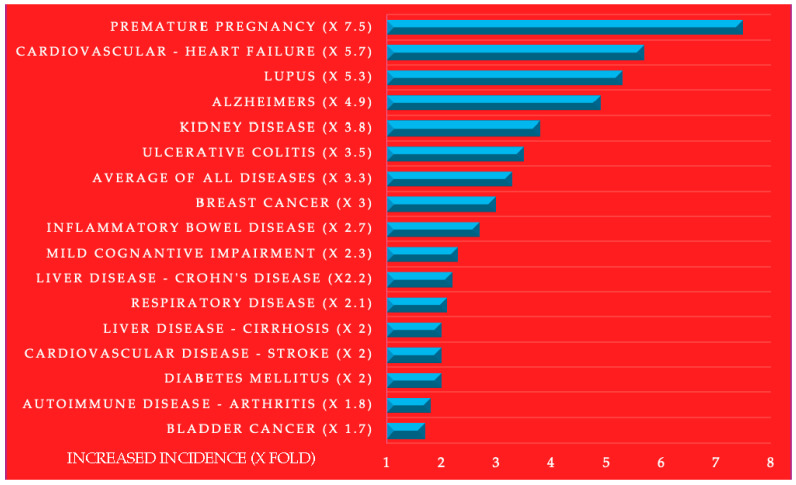
Bar chart showing the increased incidence of systemic diseases associated with oral pathogens. A review of the literature showed that oral pathogens, particularly *P. gingivalis*, are associated with an increased incidence of systemic diseases. The average increased incidence of all the diseases is increased 3.3 times when there is a severe oral pathogen infection. Most or all of these relationships are a bidirectional feedback axis, where the disease also increases the severity of the oral diseases.

Oral pathogens that have the potential to cause disease coexist with commensal microorganisms that typically do not cause disease. However, when dysbiosis occurs—disrupting the delicate balance of the oral microbiome—the overgrowth and heightened virulence of these pathogens can significantly increase the health risks and contribute to disease development. On average, oral pathogens are associated with a 3.3-fold increase in the incidence of systemic diseases, as illustrated in Figure 7.

## 29. Oral Pathogens’ Mediators of Infection and Inflammation

**Figure 8:**
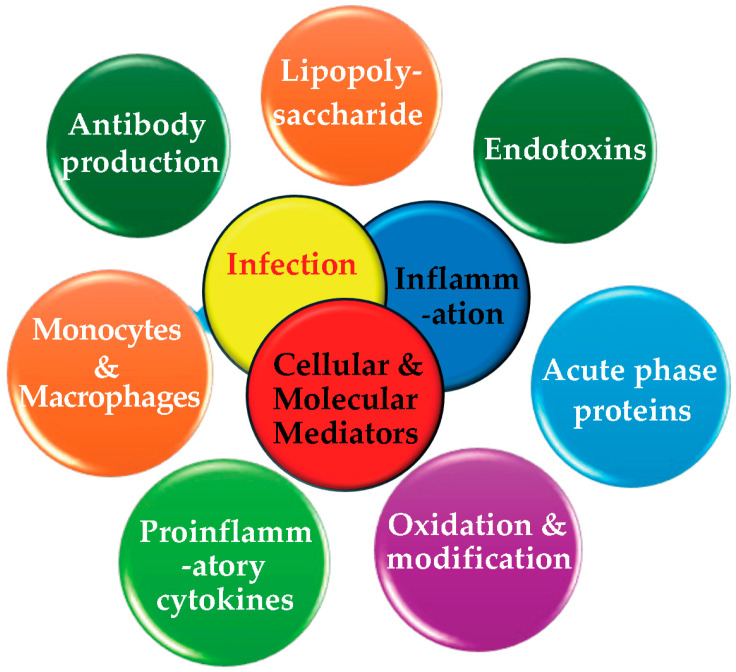
Oral pathogens’ mediators of infection and inflammation. Oral pathogens employ a range of cellular and molecular mediators, which function collectively or independently to drive infection and inflammation. These mediators differentiate pathogenic microbes, which can cause disease, from commensal microorganisms that coexist without causing harm.

Oral pathogens possess virulence genes that are activated in nutrient-rich environments, facilitating their proliferation [17,43]. These pathogens can spread locally via saliva and other bodily fluids or disseminate systemically through the bloodstream, potentially infecting distant organs [60,169]. The oral pathogens’ key mediators of infection and inflammation are shown in Figure 8

**Figure 9:**
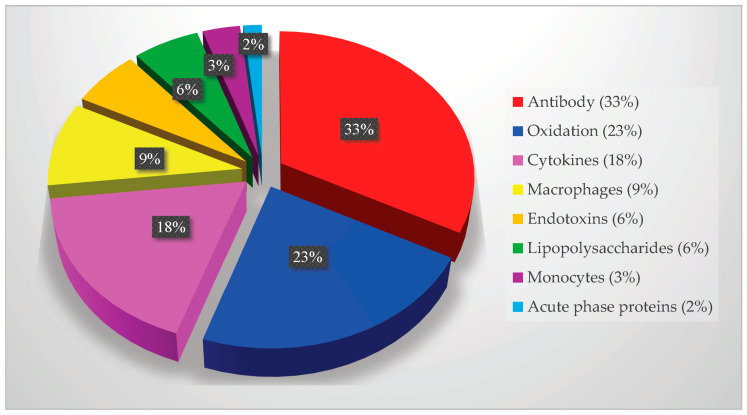
Pie chart of oral pathogens’ key mediators of infection and inflammation. The pie chart was created based on a search conducted on PubMed on 14 November 2024, with no time limit, showing the proportion of total publications available by bacteria (including oral pathogen) and the key mediators of infection and inflammation.

The immune system detects these pathogens as foreign invaders through their antigens, prompting an immune response [157,168]. Monocytes and macrophages are activated to combat the infection, while host cells release cytokines, growth factors, and acute-phase proteins to repair tissue and organ damage. The proportion of PubMed articles on oral pathogens’ key mediators of infection and inflammation are shown in Figure 9.

**Figure 10:**
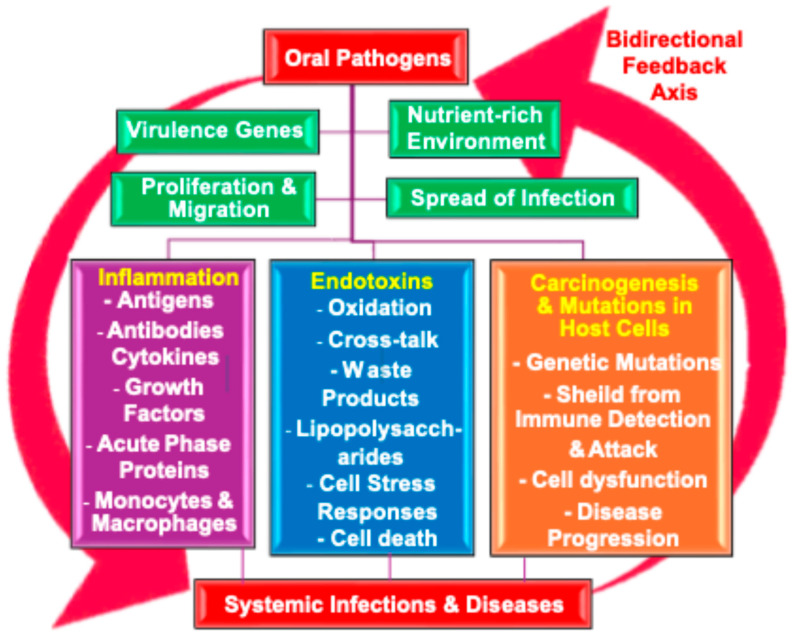
Flowchart of the oral pathogens’ disease-causing mechanisms by which oral pathogens interact with and contribute to carcinogenesis and mutations which lead to systemic diseases.

Oral pathogens secrete endotoxins such as oxidative waste products [163,183], lipopolysaccharides [75,207,233], acute phase proteins, and cytokine signaling molecules [142,143,144] to coordinate their activities with other bacteria. The endotoxins elicit inflammatory responses from antibodies [73,74,175,198,234], macrophages [52,53,78], and monocytes. Chronic inflammation will often increase the environmental stress on the host cells, potentially causing dysfunction, inefficiency, or cell death [116,177]. Furthermore, oral pathogens may contribute to carcinogenesis by inducing genetic mutations in the host cells, transforming them into cancerous or aberrant forms [21,23,62,68,132,133,134,135]. This disruption of the normal cellular functions drives disease progression. In some cases, pathogens can shield cancerous or mutated cells from immune detection, impairing the body’s ability to eliminate abnormal cells and prevent disease escalation. A flowchart illustrating the mechanisms by which oral pathogens interact with and contribute to carcinogenesis and mutations which lead to systemic diseases are shown in Figure 10.

Oral pathogens, such as *P. gingivalis*, infect a tissue, such as the periodontal (gum) tissues [19,51], and virulence genes become activated in nutrient-rich environments, enabling their proliferation [17,43]. These pathogens spread locally through saliva and other bodily fluids or disseminate systemically via the bloodstream, potentially infecting distant organs [38,54,60]. The immune system’s surveillance mechanisms detect these pathogens as foreign invaders through their antigens, triggering the activation of monocytes and macrophages to combat the infection [157,168]. In response, the host cells release cytokines, growth factors, and acute phase proteins to attempt to repair damage to tissues and organs [70,78,142,157]. But this process can disrupt the normal organ function and drive genetic mutations which cause cancer [126], dysfunction, and disease progression [136,149,152,153]. 

## 30. Treatment with Antibiotics

Approximately 10% of all antibiotics are prescribed by dentists for treating oral pathogens in dental infections [247]. Antibiotics are typically reserved for severe infections, advanced periodontitis, or cases that fail to respond to conventional mechanical treatments like scaling and root planing. The impact of antibiotic treatment on the oral microbiota depends on several factors, including the specific antibiotic used, administration method, dosage, duration of treatment, and the level of resistance that develops within the microbiota over time [248]. Commonly prescribed antibiotics for periodontitis caused by anaerobic pathogens such as *P. gingivalis* include metronidazole, amoxicillin, clindamycin, doxycycline, and ciprofloxacin [249,250]. Amoxicillin is the most frequently used antibiotic, although oral bacteria have shown a resistance rate of over 96% [251]. 

The incidence of penicillin resistance in the microflora of acute dental infections has increased significantly, from a relatively low level of 5% in abscess cases to more than 55% over the past decade [252], emphasizing the risks of over-prescribing antibiotics, which can lead to the development of drug-resistant superbugs, and pose a significant public health threat. The overuse and misuse of antibiotics accelerates the emergence of resistant bacteria, making infections harder to treat and increasing the potential for widespread, and untreatable diseases.

## 31. Conclusions

This review is long overdue as the need to understand the relationship between important chronic inflammatory diseases, such as periodontal diseases and systemic diseases, is increasing as infection disease control and public health needs also increase. This intimate relationship discussed here has not always been taken seriously in the medical community.

This review synthesizes the findings from two hundred and fifty two studies to explore the link between oral pathogens and the incidence of specific systemic diseases. The relationship between oral pathogens, particularly those associated with periodontitis, dental caries, and systemic conditions, is a bidirectional feedback axis—each dental disease can exacerbate a systemic disease, and *vice versa*. Individuals suffering from periodontitis are up to seven times more likely to develop a medical disorder or systemic disease, underscoring the profound connection between oral infections, and the ability of oral pathogens to cause a disease in other organs of the body. The oral cavity is home to over 770 microbial species, although only a subset of these species are pathogens, which are linked to systemic diseases.

Oral pathogens carry virulence genes that are activated in nutrient-rich environments, allowing them to proliferate and spread. The pathogens can disseminate locally through saliva and other bodily fluids or systemically via the bloodstream, potentially infecting distant organs. The immune system detects these pathogens as foreign invaders, triggering immune responses, including the activation of monocytes and macrophages. In response, the body releases cytokines, growth factors, and acute phase proteins to attempt to repair tissue damage.

Oral pathogens release various endotoxins, such as oxidative waste products, lipopolysaccharides, and signaling molecules, to coordinate their activities with other bacteria. The endotoxins place additional stress on the host cells, leading to dysfunction, inefficiency, or even cell death. According to the PubMed publication data, the most significant diseases associated with bacteria (including oral pathogens), ranked by the number of publications, include cancer, respiratory diseases, liver conditions, bowel diseases, fever, kidney disease, pregnancy complications, cardiovascular disease, bacteremia, diabetes, arthritis, autoimmune disorders, periodontal disease, dental caries, psychological conditions, bladder issues, dementia, lupus, and Alzheimer’s disease.

The most researched bacteria (including oral pathogens), from the most to the least are the herpes virus, *C. albicans*, *S. mutans*, *P. gingivalis*, *F. nucleatum*, *A. actinomycetemcomitans*, *P. intermedia*, *T. denticola*, and *T. forsythia*. A comparison between the disease burden caused by these oral pathogens and the proportion of related research publications reveals a significant disparity. While conditions like periodontitis, dental caries, and other oral diseases affect 26–47% of Americans, only about 1% of the scientific literature addresses these issues. This underrepresentation may result from research bias and insufficient funding for oral pathogen studies.

Increasing investment in this field could lead to substantial public health benefits, including improved health outcomes, an enhanced quality of life, and a reduction in the economic burdens of chronic diseases. Dental and periodontal treatments have been proven effective in eliminating oral infections. When combined with antibiotics, the treatments can prevent the spread of pathogens to other parts of the body, reducing the risk of systemic complications. 

Despite the decades of research, the development of oral pathogen vaccines faces significant ethical and safety challenges, particularly the risk of bioengineering antibiotic-resistant “super-microorganisms” that could exacerbate pandemics.

Caution against the overuse and misuse of antibiotics is needed, because it can accelerate the development of drug-resistant bacteria. 

Evidence supports the efficacy of dental and periodontal treatments in eliminating oral infections and reducing the severity of systemic diseases. The substantial burden that oral pathogens have on cancer, cardiovascular diseases, Alzheimer’s, diabetes, and other systemic diseases poses a significant public health crisis. 

## Data Availability

The published data are available from the articles referenced.

## Supplementary Materials

The following supporting information can be downloaded at: https://www.mdpi.com/article/10.3390/pathogens13121084/s1, The supplementary raw data file is for Figure 3, Figure 4, Figure 5, Figure 7 and Figure 9.

## References

[1] Jain N. (2020). The early life education of the immune system: Moms, microbes and (missed) opportunities. Gut Microbes.

[2] Childers N.K., Momeni S.S., Whiddon J., Cheon K., Cutter G.R., Wiener H.W., Ghazal T.S., Ruby J.D., Moser S.A. (2017). Association Between Early Childhood Caries and Colonization with *Streptococcus mutans* Genotypes From Mothers. Pediatr. Dent..

[3] Zheng D., Liwinski T., Elinav E. (2020). Interaction between microbiota and immunity in health and disease. Cell Res..

[4] Aggarwal N., Kitano S., Puah G.R.Y., Kittelmann S., Hwang I.Y., Chang M.W. (2023). Microbiome and Human Health: Current Understanding, Engineering, and Enabling Technologies. Chem. Rev..

[5] Hou K., Wu Z.X., Chen X.Y., Wang J.Q., Zhang D., Xiao C., Zhu D., Koya J.B., Wei L., Li J. (2022). Microbiota in health and diseases. Signal Transduct. Target. Ther..

[6] Kaan A.M.M., Kahharova D., Zaura E. (2021). Acquisition and establishment of the oral microbiota. Periodontology 2000.

[7] Deo P.N., Deshmukh R. (2019). Oral microbiome: Unveiling the fundamentals. J. Oral. Maxillofac. Pathol..

[8] Gao L., Xu T., Huang G., Jiang S., Gu Y., Chen F. (2018). Oral microbiomes: More and more importance in oral cavity and whole body. Protein Cell.

[9] Rapala-Kozik M., Surowiec M., Juszczak M., Wronowska E., Kulig K., Bednarek A., Gonzalez-Gonzalez M., Karkowska-Kuleta J., Zawrotniak M., Satała D. (2023). Living together: The role of *Candida albicans* in the formation of polymicrobial biofilms in the oral cavity. Yeast.

[10] Syrjänen S. (2018). Oral manifestations of human papillomavirus infections. Eur. J. Oral. Sci..

[11] Ahn J., Hayes R.B. (2021). Environmental Influences on the Human Microbiome and Implications for Noncommunicable Disease. Annu. Rev. Public. Health.

[12] Li X., Liu Y., Yang X., Li C., Song Z. (2022). The Oral Microbiota: Community Composition, Influencing Factors, Pathogenesis, and Interventions. Front. Microbiol..

[13] Huang X., Huang X., Huang Y., Zheng J., Lu Y., Mai Z., Zhao X., Cui L., Huang S. (2023). The oral microbiome in autoimmune diseases: Friend or foe?. J. Transl. Med..

[14] Sender R., Fuchs S., Milo R. (2016). Revised Estimates for the Number of Human and Bacteria Cells in the Body. PLoS Biol..

[15] Allaband C., McDonald D., Vázquez-Baeza Y., Minich J.J., Tripathi A., Brenner D.A., Loomba R., Smarr L., Sandborn W.J., Schnabl B. (2019). Microbiome 101: Studying, Analyzing, and Interpreting Gut Microbiome Data for Clinicians. Clin. Gastroenterol. Hepatol..

[16] Gilbert J.A., Blaser M.J., Caporaso J.G., Jansson J.K., Lynch S.V., Knight R. (2018). Current understanding of the human microbiome. Nat. Med..

[17] Xu W., Zhou W., Wang H., Liang S. (2020). Roles of Porphyromonas gingivalis and its virulence factors in periodontitis. Adv. Protein Chem. Struct. Biol..

[18] Mosaddad S.A., Tahmasebi E., Yazdanian A., Rezvani M.B., Seifalian A., Yazdanian M., Tebyanian H. (2019). Oral microbial biofilms: An update. Eur. J. Clin. Microbiol. Infect. Dis..

[19] Ji S., Kook J.K., Park S.N., Lim Y.K., Choi G.H., Jung J.S. (2023). Characteristics of the Salivary Microbiota in Periodontal Diseases and Potential Roles of Individual Bacterial Species To Predict the Severity of Periodontal Disease. Microbiol. Spectr..

[20] Lizárraga D., Gómez-Gil B., García-Gasca T., Ávalos-Soriano A., Casarini L., Salazar-Oroz A., García-Gasca A. (2024). Gestational diabetes mellitus: Genetic factors, epigenetic alterations, and microbial composition. Acta Diabetol..

[21] Issrani R., Reddy J., Dabah T.H.E., Prabhu N. (2022). Role of Oral Microbiota in Carcinogenesis: A Short Review. J. Cancer Prev..

[22] Grigalauskienė R., Slabšinskienė E., Vasiliauskienė I. (2015). Biological approach of dental caries management. Stomatologija.

[23] Thomas S., Izard J., Walsh E., Batich K., Chongsathidkiet P., Clarke G., Sela D.A., Muller A.J., Mullin J.M., Albert K. (2017). The Host Microbiome Regulates and Maintains Human Health: A Primer and Perspective for Non-Microbiologists. Cancer Res..

[24] Sedghi L., DiMassa V., Harrington A., Lynch S.V., Kapila Y.L. (2021). The oral microbiome: Role of key organisms and complex networks in oral health and disease. Periodontology 2000.

[25] Spatafora G., Li Y., He X., Cowan A., Tanner A.C.R. (2024). The Evolving Microbiome of Dental Caries. Microorganisms.

[26] Gopinath D., Koe K.H., Maharajan M.K., Panda S. (2023). A Comprehensive Overview of Epidemiology, Pathogenesis and the Management of Herpes Labialis. Viruses.

[27] Dreyfus D.H. (2013). Herpesviruses and the microbiome. J. Allergy Clin. Immunol..

[28] Rizzo R. (2020). Controversial role of herpesviruses in Alzheimer’s disease. PLoS Pathog..

[29] Khalesi Z., Tamrchi V., Razizadeh M.H., Letafati A., Moradi P., Habibi A., Habibi N., Heidari J., Noori M., Nahid Samiei M. (2023). Association between human herpesviruses and multiple sclerosis: A systematic review and meta-analysis. Microb. Pathog..

[30] Cohen J.I. (2024). Therapeutic vaccines for herpesviruses. J. Clin. Investig..

[31] Grose C. (2012). Pangaea and the Out-of-Africa Model of Varicella-Zoster Virus Evolution and Phylogeography. J. Virol..

[32] Arya N.R., Rafiq N.B. (2024). Candidiasis. StatPearls [Internet].

[33] Stenderup A. (1990). Oral mycology. Acta Odontol. Scand..

[34] Pavlova A., Sharafutdinov I. (2020). Recognition of *Candida albicans* and Role of Innate Type 17 Immunity in Oral Candidiasis. Microorganisms.

[35] Baker J.L., Bor B., Agnello M., Shi W., He X. (2017). Ecology of the Oral Microbiome: Beyond Bacteria. Trends Microbiol..

[36] Kaur G., Chawla S., Kumar P., Singh R. (2023). Advancing Vaccine Strategies against Candida Infections. Explor. New Front. Vaccines.

[37] Allison D.L., Willems H.M.E., Jayatilake J.A.M.S., Bruno V.M., Peters B.M., Shirtliff M.E. (2016). Candida-Bacteria Interactions: Their Impact on Human Disease. Microbiol. Spectr..

[38] Wiederhold N.P. (2017). Antifungal resistance: Current trends and future strategies to combat. Infect. Drug Resist..

[39] Esberg A., Sheng N., Mårell L., Claesson R., Persson K., Borén T., Strömberg N. (2017). Streptococcus Mutans Adhesin Biotypes that Match and Predict Individual Caries Development. EBioMedicine.

[40] Du Q., Fu M., Zhou Y., Cao Y., Guo T., Zhou Z., Li M., Peng X., Zheng X., Li Y. (2020). Sucrose promotes caries progression by disrupting the microecological balance in oral biofilms: An in vitro study. Sci. Rep..

[41] Wang Y., Hoffmann J.P., Baker S.M., Bentrup K.H.Z., Wimley W.C., Fuselier J.A., Bitoun J.P., Morici L.A. (2021). Inhibition of Streptococcus mutans biofilms with bacterial-derived outer membrane vesicles. BMC Microbiol..

[42] Zheng T., Jing M., Gong T., Yan J., Wang X., Xu M., Zhou X., Zeng J., Li Y. (2023). Regulatory mechanisms of exopolysaccharide synthesis and biofilm formation in *Streptococcus mutans*. J. Oral. Microbiol..

[43] Krzyściak W., Jurczak A., Kościelniak D., Bystrowska B., Skalniak A. (2014). The virulence of Streptococcus mutans and the ability to form biofilms. Eur. J. Clin. Microbiol. Infect. Dis..

[44] LaValley E.A., Sen S., Mason E., Logue M., Trivedi T., Moss K., Beck J., Rosamond W.D., Gottesman R.F. (2024). Dental Caries a Risk Factor for Intracerebral Hemorrhage. Cerebrovasc. Dis..

[45] Latti B.R., Kalburge J.V., Birajdar S.B., Latti R.G. (2018). Evaluation of relationship between dental caries, diabetes mellitus and oral microbiota in diabetics. J. Oral. Maxillofac. Pathol..

[46] Ucuncu M.Y., Topcuoglu N., Kulekci G., Ucuncu M.K., Erelel M., Gokce Y.B. (2024). A comparative evaluation of the effects of respiratory diseases on dental caries. BMC Oral. Health.

[47] Watanabe I., Kuriyama N., Miyatani F., Nomura R., Naka S., Nakano K., Ihara M., Iwai K., Matsui D., Ozaki E. (2016). Oral Cnm-positive Streptococcus Mutans Expressing Collagen Binding Activity is a Risk Factor for Cerebral Microbleeds and Cognitive Impairment. Sci. Rep..

[48] Durand R., Gunselman E.L., Hodges J.S., Diangelis A.J., Michalowicz B.S. (2009). A pilot study of the association between cariogenic oral bacteria and preterm birth. Oral. Dis..

[49] Liu G., Saxena D., Chen Z., Norman R.G., Phelan J.A., Laverty M., Fisch G.S., Corby P.M., Abrams W., Malamud D. (2012). HIV infection affects *Streptococcus mutans* levels, but not genotypes. J. Dent. Res..

[50] Mysak J., Podzimek S., Sommerova P., Lyuya-Mi Y., Bartova J., Janatova T., Prochazkova J., Duskova J. (2014). Porphyromonas gingivalis: Major periodontopathic pathogen overview. J. Immunol. Res..

[51] Ong G. (1998). Periodontal disease and tooth loss. Int. Dent. J..

[52] Wang H., Peng W., Zhang G., Jiang M., Zhao J., Zhao X., Pan Y., Lin L. (2022). Role of PG0192 and PG0193 in the modulation of pro-inflammatory cytokines in macrophages in response to *Porphyromonas gingivalis*. Eur. J. Oral. Sci..

[53] Fan X., Zheng S., Chen C., Lin L., Wang H., Shen Y., Pan Y., Li C. (2023). Sialidase facilitates *Porphyromonas gingivalis* immune evasion by reducing M1 polarization, antigen presentation, and phagocytosis of infected macrophages. Front. Cell Infect. Microbiol..

[54] Mahendra J., Mahendra L., Kurian V.M., Jaishankar K., Mythilli R. (2009). Prevalence of periodontal pathogens in coronary atherosclerotic plaque of patients undergoing coronary artery bypass graft surgery. J. Maxillofac. Oral. Surg..

[55] Liu F., Zhu B., An Y., Zhou Z., Xiong P., Li X., Mi Y., He T., Chen F., Wu B. (2024). Gingipain from Porphyromonas gingivalis causes insulin resistance by degrading insulin receptors through direct proteolytic effects. Int. J. Oral. Sci..

[56] Perricone C., Ceccarelli F., Saccucci M., Di Carlo G., Bogdanos D.P., Lucchetti R., Pilloni A., Valesini G., Polimeni A., Conti F. (2019). Porphyromonas gingivalis and rheumatoid arthritis. Curr. Opin. Rheumatol..

[57] Dominy S.S., Lynch C., Ermini F., Benedyk M., Marczyk A., Konradi A., Nguyen M., Haditsch U., Raha D., Griffin C. (2019). Porphyromonas gingivalis in Alzheimer’s disease brains: Evidence for disease causation and treatment with small-molecule inhibitors. Sci. Adv..

[58] Song B., Xian W., Sun Y., Gou L., Guo Q., Zhou X., Ren B., Cheng L. (2023). Akkermansia muciniphila inhibited the periodontitis caused by *Fusobacterium nucleatum*. NPJ Biofilms Microbiomes.

[59] Liu P.F., Huang I.F., Shu C.W., Huang C.M. (2013). Halitosis vaccines targeting FomA, a biofilm-bridging protein of fusobacteria nucleatum. Curr. Mol. Med..

[60] Stokowa-Sołtys K., Wojtkowiak K., Jagiełło K. (2021). Fusobacterium nucleatum—Friend or foe?. J. Inorg. Biochem..

[61] Li Z., Liu Y., Huang X., Wang Q., Fu R., Wen X., Liu J., Zhang L.F. (2024). Nucleatum enhances oral squamous cell carcinoma proliferation via E-cadherin/β-Catenin pathway. BMC Oral. Health.

[62] Li R., Shen J., Xu Y. (2022). Fusobacterium nucleatum and Colorectal Cancer. Infect. Drug Resist..

[63] Zhang L., Leng X.X., Qi J., Wang N., Han J.X., Tao Z.H., Zhuang Z.Y., Ren Y., Xie Y.L., Jiang S.S. (2024). The adhesin RadD enhances Fusobacterium nucleatum tumour colonization and colorectal carcinogenesis. Nat. Microbiol..

[64] Vander Haar E.L., So J., Gyamfi-Bannerman C., Han Y.W. (2018). Fusobacterium nucleatum and adverse pregnancy outcomes: Epidemiological and mechanistic evidence. Anaerobe.

[65] Lee H.R., Jun H.K., Kim H.D., Lee S.H., Choi B.K. (2012). Fusobacterium nucleatum GroEL induces risk factors of atherosclerosis in human microvascular endothelial cells and ApoE(-/-) mice. Mol. Oral. Microbiol..

[66] Borsa L., Dubois M., Sacco G., Lupi L. (2021). Analysis the Link between Periodontal Diseases and Alzheimer’s Disease: A Systematic Review. Int. J. Env. Res. Public Health.

[67] Yan C., Diao Q., Zhao Y., Zhang C., He X., Huang R., Li Y. (2022). Fusobacterium nucleatum infection-induced neurodegeneration and abnormal gut microbiota composition in Alzheimer’s disease-like rats. Front. Neurosci..

[68] Jiang S.S., Xie Y.L., Xiao X.Y., Kang Z.R., Lin X.L., Zhang L., Li C.S., Qian Y., Xu P.P., Leng X.X. (2023). Fusobacterium nucleatum-derived succinic acid induces tumor resistance to immunotherapy in colorectal cancer. Cell Host Microbe.

[69] Christersson L.A. (1993). Actinobacillus actinomycetemcomitans and localized juvenile periodontitis. Clinical, microbiologic and histologic studies. Swed. Dent. J. Suppl..

[70] Gholizadeh P., Pormohammad A., Eslami H., Shokouhi B., Fakhrzadeh V., Kafil H.S. (2017). Oral pathogenesis of Aggregatibacter actinomycetemcomitans. Microb. Pathog..

[71] Johansson A. (2011). Aggregatibacter actinomycetemcomitans leukotoxin: A powerful tool with capacity to cause imbalance in the host inflammatory response. Toxins.

[72] Liljestrand J.M., Paju S., Pietiäinen M., Buhlin K., Persson G.R., Nieminen M.S., Sinisalo J., Mäntylä P., Pussinen P.J. (2018). Immunologic burden links periodontitis to acute coronary syndrome. Atherosclerosis.

[73] Colhoun H.M., Slaney J.M., Rubens M.B., Fuller J.H., Sheiham A., Curtis M.A. (2008). Antibodies to periodontal pathogens and coronary artery calcification in type 1 diabetic and nondiabetic subjects. J. Periodontal Res..

[74] Ogrendik M., Kokino S., Ozdemir F., Bird P.S., Hamlet S. (2005). Serum antibodies to oral anaerobic bacteria in patients with rheumatoid arthritis. MedGenMed.

[75] Fan X., Alekseyenko A.V., Wu J., Peters B.A., Jacobs E.J., Gapstur S.M., Purdue M.P., Abnet C.C., Stolzenberg-Solomon R., Miller G. (2018). Human oral microbiome and prospective risk for pancreatic cancer: A population-based nested case-control study. Gut.

[76] Arcuri C., Petro E., Sollecchia G., Mummolo S., Marzo G. (2020). Laser in periodontal pockets: In vivo and in vitro study. J. Biol. Regul. Homeost. Agents.

[77] Söder B., Källmén H., Yucel-Lindberg T., Meurman J.H. (2021). Periodontal microorganisms and diagnosis of malignancy: A cross-sectional study. Tumour Biol..

[78] Qin Y., Li Z., Liu T., Ma J., Liu H., Zhou Y., Wang S., Zhang L., Peng Q., Ye P. (2024). Prevotella intermedia boosts OSCC progression through ISG15 upregulation: A new target for intervention. J. Cancer Res. Clin. Oncol..

[79] Lo C.H., Wu D.C., Jao S.W., Wu C.C., Lin C.Y., Chuang C.H., Lin Y.B., Chen C.H., Chen Y.T., Chen J.H. (2022). Enrichment of Prevotella intermedia in human colorectal cancer and its additive effects with Fusobacterium nucleatum on the malignant transformation of colorectal adenomas. J. Biomed. Sci..

[80] Takeuchi Y., Umeda M., Sakamoto M., Benno Y., Huang Y., Ishikawa I. (2001). *Treponema socranskii*, *Treponema denticola*, and *Porphyromonas gingivalis* are associated with severity of periodontal tissue destruction. J. Periodontol..

[81] Simonson L.G., Goodman C.H., Bial J.J., Morton H.E. (1988). Quantitative relationship of Treponema denticola to severity of periodontal disease. Infect. Immun..

[82] Smajs D., Norris S.J., Weinstock G.M. (2012). Genetic diversity in Treponema pallidum: Implications for pathogenesis, evolution and molecular diagnostics of syphilis and yaws. Infect. Genet. Evol..

[83] Pisani F., Pisani V., Arcangeli F., Harding A., Singhrao S.K. (2022). The Mechanistic Pathways of Periodontal Pathogens Entering the Brain: The Potential Role of Treponema denticola in Tracing Alzheimer’s Disease Pathology. Int. J. Env. Res. Public Health.

[84] Nieminen M.T., Listyarifah D., Hagström J., Haglund C., Grenier D., Nordström D., Uitto V.J., Hernandez M., Yucel-Lindberg T., Tervahartiala T. (2018). Treponema denticola chymotrypsin-like proteinase may contribute to orodigestive carcinogenesis through immunomodulation. Br. J. Cancer.

[85] Inaba H., Amano A. (2010). Roles of oral bacteria in cardiovascular diseases—From molecular mechanisms to clinical cases: Implication of periodontal diseases in development of systemic diseases. J. Pharmacol. Sci..

[86] Bodet C., Grenier D. (2010). Synergistic effects of lipopolysaccharides from periodontopathic bacteria on pro-inflammatory cytokine production in an ex vivo whole blood model. Mol. Oral. Microbiol..

[87] Schäffer C., Andrukhov O. (2024). The intriguing strategies of Tannerella forsythia’s host interaction. Front. Oral. Health.

[88] Reis A.A., Monteiro M.F., Bonilha G.M., Saraiva L., Araújo C., Santamaria M.P., Casati M.Z., Kumar P., Casarin R.C.V. (2023). Parents with periodontitis drive the early acquisition of dysbiotic microbiomes in their offspring. J. Clin. Periodontol..

[89] Kouanda B., Sattar Z., Geraghty P. (2021). Periodontal Diseases: Major Exacerbators of Pulmonary Diseases?. Pulm. Med..

[90] Koga A., Ariyoshi W., Kobayashi K., Izumi M., Isobe A., Akifusa S., Nishihara T. (2022). The Association between Tannerella forsythia and the Onset of Fever in Older Nursing Home Residents: A Prospective Cohort Study. Int. J. Env. Res. Public Health.

[91] Wadhawan A., Reynolds M.A., Makkar H., Scott A.J., Potocki E., Hoisington A.J., Brenner L.A., Dagdag A., Lowry C.A., Dwivedi Y. (2020). Periodontal Pathogens and Neuropsychiatric Health. Curr. Top. Med. Chem..

[92] Buttorff C., Ruder T., Bauman M. (2017). Multiple Chronic Conditions in the United States.

[93] National Health Expenditure Data: Historical. Center for Medicare & Medicaid Services. Updated 13 December 2023. https://www.cms.gov/data-research/statistics-trends-and-reports/national-health-expenditure-data/historical.

[94] Centers for Disease Control and Prevention, National Center for Health Statistics Multiple Cause of Death 2018–2022 on CDC WONDER Online Database website. http://wonder.cdc.gov/mcd.html.

[95] Centers for Disease Control and Prevention, Fast Facts: Health and Economic Costs of Chronic Conditions. https://www.cdc.gov/chronic-disease/data-research/facts-stats/index.html.

[96] Cancer Statistics Working Group U.S. Cancer Statistics Data Visualizations Tool, Based on 2021 Submission Data (1999–2021): U.S. Department of Health and Human Services, Centers for Disease Control and Prevention, and National Cancer Institute. Updated June 2024. www.cdc.gov/cancer/dataviz.

[97] Centers for Disease Control and Prevention (2023). Diabetes Statistics Report. U.S. Dept of Health and Human Services. https://www.cdc.gov/diabetes/php/data-research/index.html.

[98] Hippisley-Cox J., Coupland C. (2016). Diabetes treatments and risk of amputation, blindness, severe kidney failure, hyperglycaemia, and hypoglycaemia: Open cohort study in primary care. BMJ.

[99] Consolaro A. (2013). In adults: 47.2% have periodontitis! How about in orthodontic patients?. Dent. Press. J. Orthod..

[100] U.S. Department of Health and Human Services, Centers for Disease Control and Prevention, Prevalence of Obesity and Severe Obesity Among Adults: United States, 2017–2018. NCHS Data Brief No. 360, February 2020. https://www.cdc.gov/nchs/data/databriefs/db360-h.pdf.

[101] National Cancer Institute Cancer Statistics. https://www.cancer.gov/about-cancer/understanding/statistics.

[102] Kazi D.S., Elkind M.S.V., Deutsch A., Dowd W.N., Heidenreich P., Khavjou O., Mark D., Mussolino M.E., Ovbiagele B., Patel S.S. (2024). Forecasting the Economic Burden of Cardiovascular Disease and Stroke in the United States Through 2050: A Presidential Advisory From the American Heart Association. Circulation.

[103] National Institutes of Dental & Craniofacial Research Dental Caries (Tooth Decay) in Adults (Ages 20 to 64 Years). https://www.nidcr.nih.gov/research/data-statistics/dental-caries/adults.

[104] Fallon E.A., Boring M.A., Foster A.L., Stowe E.W., Lites T.D., Odom E.L., Seth P. (2023). Prevalence of Diagnosed Arthritis—United States, 2019–2021. MMWR Morb Mortal Wkly Rep..

[105] Centers for Disease Control and Prevention (2023). Chronic Kidney Disease in the United States. https://www.cdc.gov/kidney-disease/php/data-research/.

[106] Centers for Disease Control and Prevention, National Center for Health Statistics. https://www.cdc.gov/nchs/fastats/asthma.htm.

[107] Sacks D.B., Arnold M., Bakris G.L., Bruns D.E., Horvath A.R., Lernmark Å., Metzger B.E., Nathan D.M., Kirkman M.S. (2023). Guidelines and Recommendations for Laboratory Analysis in the Diagnosis and Management of Diabetes Mellitus. Diabetes Care.

[108] Alzheimer’s Association (2024). 2024 Alzheimer’s disease facts and figures. Alzheimers Dement..

[109] Fairweather D., Frisancho-Kiss S., Rose N.R. (2008). Sex differences in autoimmune disease from a pathological perspective. Am. J. Pathol..

[110] Armstrong A.W., Mehta M.D., Schupp C.W., Gondo G.C., Bell S.J., Griffiths C.E.M. (2021). Psoriasis Prevalence in Adults in the United States. JAMA Dermatol..

[111] Chronic Liver Disease and Cirrhosis, National Center for Health Statistics. https://www.cdc.gov/nchs/fastats/liver-disease.htm.

[112] Lewis J.D., Parlett L.E., Jonsson Funk M.L., Brensinger C., Pate V., Wu Q., Dawwas G.K., Weiss A., Constant B.D., McCauley M. (2023). Incidence, Prevalence, and Racial and Ethnic Distribution of Inflammatory Bowel Disease in the United States. Gastroenterology.

[113] Kinane D.F., Stathopoulou P.G., Papapanou P.N. (2017). Periodontal diseases. Nat. Rev. Dis. Primers.

[114] Peng X., Cheng L., You Y., Tang C., Ren B., Li Y., Xu X., Zhou X. (2022). Oral microbiota in human systematic diseases. Int. J. Oral. Sci..

[115] Poulsen C.S., Nygaard N., Constancias F., Stankevic E., Kern T., Witte D.R., Vistisen D., Grarup N., Pedersen O.B., Belstrøm D. (2022). Association of general health and lifestyle factors with the salivary microbiota—Lessons learned from the ADDITION-PRO cohort. Front. Cell Infect. Microbiol..

[116] Thoden van Velzen S.K., Abraham-Inpijn L., Moorer W.R. (1984). Plaque and systemic disease: A reappraisal of the focal infection concept. J. Clin. Periodontol..

[117] Jaramillo A., Lafaurie G.I., Millán L.V., Ardila C.M., Duque A., Novoa C., López D., Contreras A. (2013). Association between periodontal disease and plasma levels of cholesterol and triglycerides. Colomb. Med..

[118] Humphrey L.L., Fu R., Buckley D.I., Freeman M., Helfand M. (2008). Periodontal disease and coronary heart disease incidence: A systematic review and meta-analysis. J. Gen. Intern. Med..

[119] Leira Y., Seoane J., Blanco M., Rodríguez-Yáñez M., Takkouche B., Blanco J., Castillo J. (2017). Association between periodontitis and ischemic stroke: A systematic review and meta-analysis. Eur. J. Epidemiol..

[120] Lockhart P.B., Bolger A.F., Papapanou P.N., Osinbowale O., Trevisan M., Levison M.E., Taubert K.A., Newburger J.W., Gornik H.L., Gewitz M.H. (2012). Periodontal disease and atherosclerotic vascular disease: Does the evidence support an independent association?: A scientific statement from the American Heart Association. Circulation.

[121] Ketelhuth D.F., Hansson G.K. (2016). Adaptive response of T and B cells in atherosclerosis. Circ. Res..

[122] Zemedikun D.T., Chandan J.S., Raindi D., Rajgor A.D., Gokhale K.M., Thomas T., Falahee M., De Pablo P., Lord J.M., Raza K. (2021). Burden of chronic diseases associated with periodontal diseases: A retrospective cohort study using UK primary care data. BMJ Open.

[123] Falcao A., Bullón P. (2019). A review of the influence of periodontal treatment in systemic diseases. Periodontology 2000.

[124] Hajishengallis G. (2022). Interconnection of periodontal disease and comorbidities: Evidence, mechanisms, and implications. Periodontology 2000.

[125] Patel M. (2020). Dental caries vaccine: Are we there yet?. Lett. Appl. Microbiol..

[126] Sfreddo C.S., Maier J., De David S.C., Susin C., Moreira C.H.C. (2017). Periodontitis and breast cancer: A case-control study. Community Dent. Oral. Epidemiol..

[127] Jia G., Zhi A., Lai P.F.H., Wang G., Xia Y., Xiong Z., Zhang H., Che N., Ai L. (2018). The oral microbiota—A mechanistic role for systemic diseases. Br. Dent. J..

[128] Janati A.I., Durand R., Karp I., Voyer R., Latulippe J.F., Emami E. (2016). Association between oral conditions and colorectal cancer: A literature review and synthesis. Rev. Epidemiol. Sante Publique.

[129] Flynn K.J., Baxter N.T., Schloss P.D. (2016). Metabolic and community synergy of oral bacteria in colorectal cancer. mSphere.

[130] Gallimidi A.B., Fischman S., Revach B., Bulvik R., Rubinstein A.M., Nussbaum G., Elkin M. (2015). Periodontal pathogens *Porphyromonas gingivalis* and *Fusobacterium nucleatum* promote tumor progression in an oral-specific chemical carcinogenesis model. Oncotarget.

[131] Makkawi H., Hoch S., Burns E., Hosur K., Hajishengallis G., Kirschning C.J., Nussbaum G. (2017). Porphyromonas gingivalis Stimulates TLR2-PI3K Signaling to escape immune clearance and induce bone resorption independently of MyD88. Front. Cell. Infect. Microbiol..

[132] Nosho K., Sukawa Y., Adachi Y., Ito M., Mitsuhashi K., Kurihara H., Kanno S., Yamamoto I., Ishigami K., Igarashi H. (2016). Association of Fusobacterium nucleatum with immunity and molecular alterations in colorectal cancer. World J. Gastroenterol..

[133] Gur C., Ibrahim Y., Isaacson B., Yamin R., Abed J., Gamliel M., Enk J., Bar-On Y., Stanietsky-Kaynan N., Coppenhagen-Glazer S. (2015). Binding of the Fap2 protein of Fusobacterium nucleatum to human inhibitory receptor TIGIT protects tumours from immune cell attack. Immunity.

[134] Sun J., Tang Q., Yu S., Xie M., Xie Y., Chen G., Chen L. (2020). Role of the oral microbiota in cancer evolution and progression. Cancer Med..

[135] Kageyama S., Takeshita T., Takeuchi K., Asakawa M., Matsumi R., Furuta M., Shibata Y., Nagai K., Ikebe M., Morita M. (2019). Characteristics of the Salivary Microbiota in Patients With Various Digestive Tract Cancers. Front. Microbiol..

[136] Scales B.S., Erb-Downward J.R., Huffnagle I.M., Lipuma J.J., Huffnagle G.B. (2015). Comparative genomics of Pseudomonas fluorescens subclade III strains from human lungs. BMC Genom..

[137] Dickson R.P., Erbdownward J.R., Huffnagle G.B. (2015). Homeostasis and its disruption in the lung microbiome. Am. J. Physiol. Lung Cell Mol. Physiol..

[138] Dickson R.P., Erbdownward J.R., Martinez F.J., Huffnagle G.B. (2016). The microbiome and the respiratory tract. Physiology.

[139] Gleeson K., Eggli D.F., Maxwell S.L. (1997). Quantitative aspiration during sleep in normal subjects. Chest.

[140] Segal L.N., Alekseyenko A.V., Clemente J.C., Kulkarni R., Wu B., Gao Z., Chen H., Berger K.I., Goldring R.M., Rom W.N. (2013). Enrichment of lung microbiome with supraglottic taxa is associated with increased pulmonary inflammation. Microbiome.

[141] Durack J., Lynch S.V., Nariya S., Bhakta N.R., Beigelman A., Castro M., Dyer A.M., Israel E., Kraft M., Martin R.J. (2017). National Heart, Lung and Blood Institute’s “AsthmaNet”. Features of the bronchial bacterial microbiome associated with atopy, asthma, and responsiveness to inhaled corticosteroid treatment. J. Allergy Clin. Immunol..

[142] Aaron S.D., Angel J.B., Lunau M., Wright K., Fex C., Le Saux N., Dales R.E. (2001). Granulocyte inflammatory markers and airway infection during acute exacerbation of chronic obstructive pulmonary disease. Am. J. Respir. Crit. Care Med..

[143] Thomas P.S. (2001). Tumour necrosis factor-alpha: The role of this multifunctional cytokine in asthma. Immunol. Cell Biol..

[144] Jousilahti P., Salomaa V., Hakala K., Rasi V., Vahtera E., Palosuo T. (2002). The association of sensitive systemic inflammation markers with bronchial asthma. Ann. Allergy Asthma Immunol..

[145] Shi T., Wang J., Dong J., Hu P., Guo Q. (2023). Periodontopathogens Porphyromonas gingivalis and Fusobacterium nucleatum and Their Roles in the Progression of Respiratory Diseases. Pathogens.

[146] de Steenhuijsen Piters W.A., Huijskens E.G., Wyllie A.L., Biesbroek G., van den Bergh M.R., Veenhoven R.H., Wang X., Trzciński K., Bonten M.J., Rossen J.W. (2016). Dysbiosis of upper respiratory tract microbiota in elderly pneumonia patients. ISME J..

[147] Salk H.M., Simon W.L., Lambert N.D., Kennedy R.B., Grill D.E., Kabat B.F., Poland G.A. (2016). Taxa of the Nasal Microbiome Are Associated with Influenza-Specific IgA Response to Live Attenuated Influenza Vaccine. PLoS ONE.

[148] Irani S., Schmidlin P.R., Bolivar I., Speich R., Boehler A. (2011). Evidence for graft colonization with periodontal pathogens in lung transplant recipients. A pilot study. Schweiz. Mon. Zahnmed..

[149] Brook I., Frazier E.H. (2003). Immune response to *Fusobacterium nucleatum* and *Prevotella intermedia* in the sputum of patients with acute exacerbation of chronic bronchitis. Chest.

[150] Lin E.C., Chiang Y.C., Lin H.Y., Tseng S.Y., Hsieh Y.T., Shieh J.A., Huang Y.H., Tsai H.T., Feng S.W., Peng T.Y. (2023). Unraveling the Link between Periodontitis and Coronavirus Disease 2019: Exploring Pathogenic Pathways and Clinical Implications. Biomedicines.

[151] Marouf N., Cai W., Said K.N., Daas H., Diab H., Chinta V.R., Hssain A.A., Nicolau B., Sanz M., Tamimi F. (2021). Association between periodontitis and severity of COVID-19 infection: A case-control study. J. Clin. Periodontol..

[152] Ladegaard Grønkjær L., Holmstrup P., Schou S., Jepsen P., Vilstrup H. (2018). Severe periodontitis and higher cirrhosis mortality. United Eur. Gastroenterol. J..

[153] Domokos Z., Uhrin E., Szabó B., Czumbel M.L., Dembrovszky F., Kerémi B., Varga G., Hegyi P., Hermann P., Németh O. (2022). Patients with inflammatory bowel disease have a higher chance of developing periodontitis: A systematic review and meta-analysis. Front. Med..

[154] Costa F.O., Lages E.J.P., Lages E.M.B., Cota L.O.M. (2019). Periodontitis in individuals with liver cirrhosis: A case-control study. J. Clin. Periodontol..

[155] Teng M.L., Ng C.H., Huang D.Q., Chan K.E., Tan D.J., Lim W.H., Yang J.D., Tan E., Muthiah M.D. (2023). Global incidence and prevalence of nonalcoholic fatty liver disease. Clin. Mol. Hepatol..

[156] Yoneda M., Naka S., Nakano K., Wada K., Endo H., Mawatari H., Imajo K., Nomura R., Hokamura K., Ono M. (2012). Involvement of a periodontal pathogen, Porphyromonas gingivalis on the pathogenesis of non-alcoholic fatty liver disease. BMC Gastroenterol..

[157] Duan Y., Pan X., Luo J., Xiao X., Li J., Bestman P.L., Luo M. (2022). Association of Inflammatory Cytokines With Non-Alcoholic Fatty Liver Disease. Front. Immunol..

[158] Yao C., Lan D., Li X., Wang Y., Qi S., Liu Y. (2023). Porphyromonas gingivalis is a risk factor for the development of nonalcoholic fatty liver disease via ferroptosis. Microbes Infect..

[159] Wang B., Deng J., Donati V., Merali N., Frampton A.E., Giovannetti E., Deng D. (2024). The Roles and Interactions of Porphyromonas gingivalis and Fusobacterium nucleatum in Oral and Gastrointestinal Carcinogenesis: A Narrative Review. Pathogens.

[160] Cai Z., Zhu T., Liu F., Zhuang Z., Zhao L. (2021). Co-pathogens in Periodontitis and Inflammatory Bowel Disease. Front. Med..

[161] Pignatelli P., Nuccio F., Piattelli A., Curia M.C. (2023). The Role of Fusobacterium nucleatum in Oral and Colorectal Carcinogenesis. Microorganisms.

[162] Mukherjee S., Chopra A., Karmakar S., Bhat S.G. (2024). Periodontitis increases the risk of gastrointestinal dysfunction: An update on the plausible pathogenic molecular mechanisms. Crit. Rev. Microbiol..

[163] Irfan M., Delgado R.Z.R., Frias-Lopez J. (2020). The Oral Microbiome and Cancer. Front. Immunol..

[164] Elzayat H., Mesto G., Al-Marzooq F. (2023). Unraveling the Impact of Gut and Oral Microbiome on Gut Health in Inflammatory Bowel Diseases. Nutrients.

[165] Sun J., Zhou M., Salazar C.R., Hays R., Bedi S., Chen Y., Li Y. (2017). Chronic Periodontal Disease, Periodontal Pathogen Colonization, and Increased Risk of Precancerous Gastric Lesions. J. Periodontol..

[166] Xiang B., Hu J., Zhang M., Zhi M. (2024). The involvement of oral bacteria in inflammatory bowel disease. Gastroenterol. Rep..

[167] Forner L., Larsen T., Kilian M., Holmstrup P. (2006). Incidence of bacteremia after chewing, tooth brushing and scaling in individuals with periodontal inflammation. J. Clin. Periodontol..

[168] Karachaliou I.G., Karachalios G.N., Kanakis K.V., Petrogiannopoulos C.L., Zacharof A.K. (2007). Fever of unknown origin due to dental infections: Cases report and review. Am. J. Med. Sci..

[169] Horliana A.C., Chambrone L., Foz A.M., Artese H.P., Rabelo Mde S., Pannuti C.M., Romito G.A. (2014). Dissemination of periodontal pathogens in the bloodstream after periodontal procedures: A systematic review. PLoS ONE.

[170] Prajitha N., Athira S.S., Mohanan P.V. (2018). Pyrogens, a polypeptide produces fever by metabolic changes in hypothalamus: Mechanisms and detections. Immunol. Lett..

[171] Del Giudice C., Vaia E., Liccardo D., Marzano F., Valletta A., Spagnuolo G., Ferrara N., Rengo C., Cannavo A., Rengo G. (2021). Infective Endocarditis: A Focus on Oral Microbiota. Microorganisms.

[172] Kitaya S., Kanamori H., Baba H., Oshima K., Takei K., Seike I., Katsumi M., Katori Y., Tokuda K. (2023). Clinical and Epidemiological Characteristics of Persistent Bacteremia: A Decadal Observational Study. Pathogens.

[173] Martins C.C., Lockhart P.B., Firmino R.T., Kilmartin C., Cahill T.J., Dayer M., Occhi-Alexandre I.G.P., Lai H., Ge L., Thornhill M.H. (2024). Bacteremia following different oral procedures: Systematic review and meta-analysis. Oral. Dis..

[174] Grubbs V., Vittinghoff E., Beck J.D., Kshirsagar A.V., Wang W., Griswold M.E., Powe N.R., Correa A., Young B. (2015). Association Between Periodontal Disease and Kidney Function Decline in African Americans: The Jackson Heart Study. J. Periodontol..

[175] Lund Håheim L., Thelle D.S., Rønningen K.S., Olsen I., Schwarze P.E. (2022). Low level of antibodies to the oral bacterium Tannerella forsythia predicts bladder cancers and Treponema denticola predicts colon and bladder cancers: A prospective cohort study. PLoS ONE.

[176] Li L., Zhang Y.L., Liu X.Y., Meng X., Zhao R.Q., Ou L.L., Li B.Z., Xing T. (2021). Periodontitis Exacerbates and Promotes the Progression of Chronic Kidney Disease Through Oral Flora, Cytokines, and Oxidative Stress. Front. Microbiol..

[177] Kitamura M., Mochizuki Y., Miyata Y., Obata Y., Mitsunari K., Matsuo T., Ohba K., Mukae H., Yoshimura A., Nishino T. (2019). Pathological Characteristics of Periodontal Disease in Patients with Chronic Kidney Disease and Kidney Transplantation. Int. J. Mol. Sci..

[178] Ariyamuthu V.K., Nolph K.D., Ringdahl B.E. (2013). Periodontal disease in chronic kidney disease and end-stage renal disease patients: A review. Cardiorenal Med..

[179] Delbove T., Gueyffier F., Juillard L., Kalbacher E., Maucort-Boulch D., Nony P., Grosgogeat B., Gritsch K. (2021). Effect of periodontal treatment on the glomerular filtration rate, reduction of inflammatory markers and mortality in patients with chronic kidney disease: A systematic review. PLoS ONE.

[180] Maringhini S., Zoccali C. (2024). Chronic Kidney Disease Progression-A Challenge. Biomedicines.

[181] Offenbacher S., Lin D., Strauss R., McKaig R., Irving J., Barros S.P., Moss K., Barrow D.A., Hefti A., Beck J.D. (2006). Effects of periodontal therapy during pregnancy on periodontal status, biologic parameters, and pregnancy outcomes: A pilot study. J. Periodontol..

[182] Vidmar Šimic M., Maver A., Zimani A.N., Hočevar K., Peterlin B., Kovanda A., Premru-Sršen T. (2023). Oral microbiome and preterm birth. Front. Med..

[183] Shira Davenport E. (2010). Preterm low birthweight and the role of oral bacteria. J. Oral. Microbiol..

[184] Fujiwara N., Tsuruda K., Iwamoto Y., Kato F., Odaki T., Yamane N., Hori Y., Harashima Y., Sakoda A., Tagaya A. (2015). Significant increase of oral bacteria in the early pregnancy period in Japanese women. J. Investig. Clin. Dent..

[185] Jensen J., Liljemark W., Bloomquist C. (1981). The effect of female sex hormones on subgingival plaque. J. Periodontol..

[186] Starzyńska A., Wychowański P., Nowak M., Sobocki B.K., Jereczek-Fossa B.A., Słupecka-Ziemilska M. (2022). Association between Maternal Periodontitis and Development of Systematic Diseases in Offspring. Int. J. Mol. Sci..

[187] Le Q.A., Akhter R., Coulton K.M., Vo N.T.N., Duong L.T.Y., Nong H.V., Yaacoub A., Condous G., Eberhard J., Nanan R. (2022). Periodontitis and Preeclampsia in Pregnancy: A Systematic Review and Meta-Analysis. Matern. Child. Health J..

[188] Tsikouras P., Oikonomou E., Nikolettos K., Andreou S., Kyriakou D., Damaskos C., Garmpis N., Monastiridou V., Nalmpanti T., Bothou A. (2024). The Impact of Periodontal Disease on Preterm Birth and Preeclampsia. J. Pers. Med..

[189] Damle S.G., Yadav R., Garg S., Dhindsa A., Beniwal V., Loomba A., Chatterjee S. (2016). Transmission of mutans streptococci in mother-child pairs. Indian. J. Med. Res..

[190] Bendek M.J., Canedo-Marroquín G., Realini O., Retamal I.N., Hernández M., Hoare A., Busso D., Monteiro L.J., Illanes S.E., Chaparro A. (2021). Periodontitis and Gestational Diabetes Mellitus: A Potential Inflammatory Vicious Cycle. Int. J. Mol. Sci..

[191] Jahan S.S., Hoque Apu E., Sultana Z.Z., Islam M.I., Siddika N. (2022). Oral Healthcare during Pregnancy: Its Importance and Challenges in Lower-Middle-Income Countries (LMICs). Int. J. Env. Res. Public Health.

[192] Sanz M., Marco Del Castillo A., Jepsen S., Gonzalez-Juanatey J.R., D’Aiuto F., Bouchard P., Chapple I., Dietrich T., Gotsman I., Graziani F. (2020). Periodontitis and cardiovascular diseases: Consensus report. J. Clin. Periodontol..

[193] Yan Y., Mao M., Li Y.Q., Chen Y.J., Yu H.D., Xie W.Z., Huang Q., Leng W.D., Xiong J. (2022). Periodontitis Is Associated With Heart Failure: A Population-Based Study (NHANES III). Front. Physiol..

[194] Bourgeois D., Inquimbert C., Ottolenghi L., Carrouel F. (2019). Periodontal Pathogens as Risk Factors of Cardiovascular Diseases, Diabetes, Rheumatoid Arthritis, Cancer, and Chronic Obstructive Pulmonary Disease-Is There Cause for Consideration?. Microorganisms.

[195] Aarabi G., Eberhard J., Reissmann D.R., Heydecke G., Seedorf U. (2015). Interaction between periodontal disease and atherosclerotic vascular disease--Fact or fiction?. Atherosclerosis.

[196] Andriankaja O., Trevisan M., Falkner K., Dorn J., Hovey K., Sarikonda S., Mendoza T., Genco R. (2011). Association between periodontal pathogens and risk of nonfatal myocardial infarction. Community Dent. Oral. Epidemiol..

[197] Aoki S., Hosomi N., Nishi H., Nakamori M., Nezu T., Shiga Y., Kinoshita N., Ueno H., Ishikawa K., Imamura E. (2020). Serum IgG titers to periodontal pathogens predict 3-month outcome in ischemic stroke patients. PLoS ONE.

[198] Hanaoka Y., Soejima H., Yasuda O., Nakayama H., Nagata M., Matsuo K., Shinohara M., Izumi Y., Ogawa H. (2013). Level of serum antibody against a periodontal pathogen is associated with atherosclerosis and hypertension. Hypertens. Res..

[199] Miyatani F., Kuriyama N., Watanabe I., Nomura R., Nakano K., Matsui D., Ozaki E., Koyama T., Nishigaki M., Yamamoto T. (2015). Relationship between Cnm-positive Streptococcus mutans and cerebral microbleeds in humans. Oral. Dis..

[200] DeStefano F., Anda R.F., Kahn H.S., Williamson D.F., Russell C.M. (1993). Dental disease and risk of coronary heart disease and mortality. BMJ.

[201] Tonomura S., Ihara M., Kawano T., Tanaka T., Okuno Y., Saito S., Friedland R.P., Kuriyama N., Nomura R., Watanabe Y. (2016). Intracerebral haemorrhage and deep microbleeds associated with cnm-positive Streptococcus mutans; a hospital cohort study. Sci. Rep..

[202] Teeuw W.J., Slot D.E., Susanto H., Gerdes V.E., Abbas F., D’Aiuto F., Kastelein J.J., Loos B.G. (2014). Treatment of periodontitis improves the atherosclerotic profile: A systematic review and meta-analysis. J. Clin. Periodontol..

[203] Schenkein H.A., Papapanou P.N., Genco R., Sanz M. (2020). Mechanisms underlying the association between periodontitis and atherosclerotic disease. Periodontology 2000.

[204] Casanova L., Hughes F.J., Preshaw P.M. (2014). Diabetes and periodontal disease: A two-way relationship. Br. Dent. J..

[205] Preshaw P.M., Alba A.L., Herrera D., Jepsen S., Konstantinidis A., Makrilakis K., Taylor R. (2012). Periodontitis and diabetes: A two-way relationship. Diabetologia.

[206] American Diabetes Association (2009). Diagnosis and classification of diabetes mellitus. Diabetes Care.

[207] de Jongh C.A., de Vries T.J., Bikker F.J., Gibbs S., Krom B.P. (2023). Mechanisms of Porphyromonas gingivalis to translocate over the oral mucosa and other tissue barriers. J. Oral. Microbiol..

[208] van Eeden W.A., van Hemert A.M., Carlier I.V.E., Penninx B.W.J.H., Lamers F., Fried E.I., Schoevers R., Giltay E.J. (2020). Basal and LPS-stimulated inflammatory markers and the course of individual symptoms of depression. Transl. Psychiatry.

[209] Martyn J.A., Kaneki M., Yasuhara S. (2008). Obesity-induced insulin resistance and hyperglycemia: Etiologic factors and molecular mechanisms. Anesthesiology.

[210] Zhao D., Sun Y., Li X., Wang X., Lu L., Li C., Pan Y., Wang S. (2023). Association between Periodontitis and HbA1c Levels in Non-Diabetic Patients: A Systematic Review and Meta-Analysis. Healthcare.

[211] Jain A., Chawla M., Kumar A., Chawla R., Grover V., Ghosh S., Pandit N., Chawla P. (2020). Management of periodontal disease in patients with diabetes- good clinical practice guidelines: A joint statement by Indian Society of Periodontology and Research Society for the Study of Diabetes in India. J. Indian. Soc. Periodontol..

[212] Zhao M., Xie Y., Gao W., Li C., Ye Q., Li Y. (2023). Diabetes mellitus promotes susceptibility to periodontitis-novel insight into the molecular mechanisms. Front. Endocrinol..

[213] Barutta F., Bellini S., Durazzo M., Gruden G. (2022). Novel Insight into the Mechanisms of the Bidirectional Relationship between Diabetes and Periodontitis. Biomedicines.

[214] Spampinato S.F., Caruso G.I., De Pasquale R., Sortino M.A., Merlo S. (2020). The Treatment of Impaired Wound Healing in Diabetes: Looking among Old Drugs. Pharmaceuticals.

[215] Tang B., Yan C., Shen X., Li Y. (2022). The bidirectional biological interplay between microbiome and viruses in periodontitis and type-2 diabetes mellitus. Front. Immunol..

[216] Marchesan J.T., Gerow E.A., Schaff R., Taut A.D., Shin S.Y., Sugai J., Brand D., Burberry A., Jorns J., Lundy S.K. (2013). Porphyromonas gingivalis oral infection exacerbates the development and severity of collagen-induced arthritis. Arthritis Res. Ther..

[217] Zhou N., Zou F., Cheng X., Huang Y., Zou H., Niu Q., Qiu Y., Shan F., Luo A., Teng W. (2021). Porphyromonas gingivalis induces periodontitis, causes immune imbalance, and promotes rheumatoid arthritis. J. Leukoc. Biol..

[218] de Pablo P., Dietrich T., McAlindon T.E. (2008). Association of periodontal disease and tooth loss with rheumatoid arthritis in the US population. J. Rheumatol..

[219] Burgos R., Ordoñez G., Vázquez-Mellado J., Pineda B., Sotelo J. (2015). Occasional presence of herpes viruses in synovial fluid and blood from patients with rheumatoid arthritis and axial spondyloarthritis. Clin. Rheumatol..

[220] Fu T.C., Lin J.R., Chang C.M. (2024). Association Between Herpes Simplex Virus II Infection and Rheumatoid Arthritis in US Adults: A Population-Based Propensity Score-Matching Study. J. Clin. Rheumatol..

[221] MacGregor A.J., Snieder H., Rigby A.S., Koskenvuo M., Kaprio J., Aho K., Silman A.J. (2000). Characterizing the quantitative genetic contribution to rheumatoid arthritis using data from twins. Arthritis Rheum..

[222] Lerner A., Jeremias P., Matthias T. (2016). The World Incidence and Prevalence of Autoimmune Diseases is Increasing. Int. J. Celiac Dis..

[223] Belstrøm D., Eiberg J.M., Enevold C., Grande M.A., Jensen C.A.J., Skov L., Hansen P.R. (2020). Salivary microbiota and inflammation-related proteins in patients with psoriasis. Oral. Dis..

[224] Polak K., Bergler-Czop B., Szczepanek M., Wojciechowska K., Frątczak A., Kiss N. (2021). Psoriasis and Gut Microbiome-Current State of Art. Int. J. Mol. Sci..

[225] Zhang X., Zhang D., Jia H., Feng Q., Wang D., Liang D., Wu X., Li J., Tang L., Li Y. (2015). The oral and gut microbiomes are perturbed in rheumatoid arthritis and partly normalized after treatment. Nat. Med..

[226] Ebbers M., Lübcke P.M., Volzke J., Kriebel K., Hieke C., Engelmann R., Lang H., Kreikemeyer B., Müller-Hilke B. (2018). Interplay between *P. gingivalis*, *F. nucleatum* and *A. actinomycetemcomitans* in murine alveolar bone loss, arthritis onset and progression. Sci. Rep..

[227] Bingham C.O., Moni M. (2013). Periodontal disease and rheumatoid arthritis: The evidence accumulates for complex pathobiologic interactions. Curr. Opin. Rheumatol..

[228] Khare N., Vanza B., Sagar D., Saurav K., Chauhan R., Mishra S. (2016). Nonsurgical Periodontal Therapy decreases the Severity of Rheumatoid Arthritis: A Case-control Study. J. Contemp. Dent. Pr..

[229] Parra-Torres V., Melgar-Rodríguez S., Muñoz-Manríquez C., Sanhueza B., Cafferata E.A., Paula-Lima A.C., Díaz-Zúñiga J. (2023). Periodontal bacteria in the brain-Implication for Alzheimer’s disease: A systematic review. Oral. Dis..

[230] Hu X., Zhang J., Qiu Y., Liu Z. (2021). Periodontal disease and the risk of Alzheimer’s disease and mild cognitive impairment: A systematic review and meta-analysis. Psychogeriatrics.

[231] Hyman B.T., Phelps C.H., Beach T.G., Bigio E.H., Cairns N.J., Carrillo M.C., Dickson D.W., Duyckaerts C., Frosch M.P., Masliah E. (2012). National Institute on Aging-Alzheimer’s Association guidelines for the neuropathologic assessment of Alzheimer’s disease. Alzheimers Dement..

[232] Dugger B.N., Dickson D.W. (2017). Pathology of Neurodegenerative Diseases. Cold Spring Harb. Perspect. Biol..

[233] Costa M.J.F., De Araújo I.D.T., Da Rocha Alves L., Da Silva R.L., Dos Santos Calderon P., Borges B.C.D., De Aquino Martins A.R.L., De Vasconcelos Gurgel B.C., Lins R. (2021). Relationship of Porphyromonas gingivalis and Alzheimer’s disease: A systematic review of pre-clinical studies. Clin. Oral. Investig..

[234] Franciotti R., Pignatelli P., Carrarini C., Romei F.M., Mastrippolito M., Gentile A., Mancinelli R., Fulle S., Piattelli A., Onofrj M. (2021). Exploring the Connection between Porphyromonas gingivalis and Neurodegenerative Diseases: A Pilot Quantitative Study on the Bacterium Abundance in Oral Cavity and the Amount of Antibodies in Serum. Biomolecules.

[235] Zhong H.-J., Xie H.-X., Luo X.-M., Zhang E.-H. (2020). Association between periodontitis and systemic lupus erythematosus: A meta-analysis. Lupus.

[236] Sojod B., Pidorodeski Nagano C., Garcia Lopez G.M., Zalcberg A., Dridi S.M., Anagnostou F. (2021). Systemic Lupus Erythematosus and Periodontal Disease: A Complex Clinical and Biological Interplay. J. Clin. Med..

[237] Corrêa J.D., Calderaro D.C., Ferreira G.A., Mendonça S.M., Fernandes G.R., Xiao E., Teixeira A.L., Leys E.J., Graves D.T., Silva T.A. (2017). Subgingival microbiota dysbiosis in systemic lupus erythematosus: Association with periodontal status. Microbiome.

[238] Zharkova O., Celhar T., Cravens P.D., Satterthwaite A.B., Fairhurst A.M., Davis L.S. (2017). Pathways leading to an immunological disease: Systemic lupus erythematosus. Rheumatology.

[239] Marques C.P., Maor Y., de Andrade M.S., Rodrigues V.P., Benatti B.B. (2016). Possible evidence of systemic lupus erythematosus and periodontal disease association mediated by Toll-like receptors 2 and 4. Clin. Exp. Immunol..

[240] Bunte K., Beikler T. (2019). Th17 Cells and the IL-23/IL-17 Axis in the Pathogenesis of Periodontitis and Immune-Mediated Inflammatory Diseases. Int. J. Mol. Sci..

[241] Hussain S.B., Leira Y., Zehra S.A., Botelho J., Machado V., Ciurtin C., D’Aiuto F., Orlandi M. (2022). Periodontitis and Systemic Lupus Erythematosus: A systematic review and meta-analysis. J. Periodontal Res..

[242] Zhang R., Ma H., Wang D., Zhang H. (2024). Immune-mediated inflammatory diseases and periodontal disease: A bidirectional two-sample mendelian randomization study. BMC Immunol..

[243] Suárez L.J., Garzón H., Arboleda S., Rodríguez A. (2020). Oral Dysbiosis and Autoimmunity: From Local Periodontal Responses to an Imbalanced Systemic Immunity. A Review. Front. Immunol..

[244] Gofur N.R.P., Handono K., Nurdiana N., Kalim H. (2021). Periodontal comparison on systemic lupus erythematosus patients and healthy subjects: A cross-sectional study. Pesqui. Bras. Em Odontopediatria E Clin. Integr..

[245] Patra D., Banerjee D., Ramprasad P., Roy S., Pal D., Dasgupta S. (2023). Recent insights of obesity-induced gut and adipose tissue dysbiosis in type 2 diabetes. Front. Mol. Biosci..

[246] Clemente-Suárez V.J., Redondo-Flórez L., Beltrán-Velasco A.I., Martín-Rodríguez A., Martínez-Guardado I., Navarro-Jiménez E., Laborde-Cárdenas C.C., Tornero-Aguilera J.F. (2023). The Role of Adipokines in Health and Disease. Biomedicines.

[247] Antibiotic Resistance in Dentistry. FDI World Dental Federation. https://www.fdiworlddental.org/antibiotic-resistance-dentistry.

[248] Ardila C.M., López M.A., Guzmán I.C. (2010). High resistance against clindamycin, metronidazole and amoxicillin in Porphyromonas gingivalis and Aggregatibacter actinomycetemcomitans isolates of periodontal disease. Med. Oral. Patol. Oral. Cir. Bucal.

[249] Liñares A., Sanz-Sánchez I., Dopico J., Molina A., Blanco J., Montero E. (2023). Efficacy of adjunctive measures in the non-surgical treatment of peri-implantitis: A systematic review. J. Clin. Periodontol..

[250] Teughels W., Feres M., Oud V., Martín C., Matesanz P., Herrera D. (2020). Adjunctive effect of systemic antimicrobials in periodontitis therapy: A systematic review and meta-analysis. J. Clin. Periodontol..

[251] Sebastian A., Antony P.G., Jose M., Babu A., Sebastian J., Kunnilathu A. (2019). Institutional microbial analysis of odontogenic infections and their empirical antibiotic sensitivity. J. Oral. Biol. Craniofac Res..

[252] Lewis M.A. (2008). Why we must reduce dental prescription of antibiotics: European Union Antibiotic Awareness Day. Br. Dent. J..

